# Molecular Epidemiology and Genetic Diversity of Influenza B Viruses Based on Whole‐Genome Analysis in Japan and Myanmar, 2016–2020

**DOI:** 10.1111/irv.70234

**Published:** 2026-02-09

**Authors:** Yusuke Ichikawa, Reiko Saito, Irina Chon, Wint Wint Phyu, Yadanar Kyaw, Su Mon Kyaw Win, Japanese Influenza Collaborative Study Group, Sayaka Yoshioka, Yuyang Sun, Jiaming Li, Tri Bayu Purnama, Keita Wagatsuma, Tsutomu Tamura, Hisami Watanabe, Moe Myat Aye, Swe Setk, Htay Htay Tin

**Affiliations:** ^1^ Division of International Health (Public Health), Graduate School of Medical and Dental Sciences Niigata University Niigata Japan; ^2^ Infectious Diseases Research Center of Niigata University in Myanmar (IDRC), Graduate School of Medical and Dental Sciences Niigata University Niigata Japan; ^3^ Department of Microbiology University of Medicine Magway Myanmar; ^4^ Respiratory Medicine Department Thingangyun Sanpya General Hospital Yangon Myanmar; ^5^ National Health Laboratory, Department of Medical Services Ministry of Health Yangon Myanmar

**Keywords:** B/Victoria, B/Yamagata, genetic reassortment, influenza vaccine, seasonal influenza, whole‐genome sequences

## Abstract

**Background:**

Influenza B virus (IBV) contributes to seasonal epidemics, but its molecular evolution is less defined than influenza A. We analyzed IBVs collected in Japan and Myanmar (2016–2020) to investigate lineage dynamics, reassortment, and genetic mismatch with vaccine strains.

**Methods:**

Respiratory specimens from patients with influenza‐like illness were collected in Japan (January 2016–March 2020) and Myanmar (June 2016–October 2019). Lineages were determined by RT‐PCR, isolates cultured in MDCK‐based cells, and whole‐genome sequencing performed on the Illumina iSeq 100. Phylogenetic analyses of all eight segments and hemagglutinin (HA) comparisons with WHO‐recommended vaccine strains were conducted.

**Results:**

Of 4703 specimens, 1164 were IBV‐positive: 632 B/Victoria (322 Japan and 310 Myanmar) and 532 B/Yamagata (452 Japan and 80 Myanmar). Whole‐genome sequences were obtained for 148 isolates. Despite differing seasonality, both countries showed parallel transitions: B/Yamagata predominated in 2017–2018 but disappeared after 2019, while B/Victoria shifted from clade V1A to emerging clade V1A.3 with a three–amino acid deletion in HA (Δ162–164). Two 2018 B/Victoria reassortants in Japan contained four internal genes from B/Yamagata. Multiple HA substitutions were observed among V1A.3 viruses, differing from contemporaneous vaccine strains (clade V1A.1), indicating potential antigenic mismatch. Mutations related to antiviral resistance in polymerase acidic and neuraminidase genes were also assessed.

**Conclusions:**

This study reveals synchronized lineage shifts, inter‐lineage reassortment, and vaccine mismatches of IBVs in two distinct countries. The disappearance of B/Yamagata and emergence of divergent B/Victoria clades highlight the need for sustained molecular surveillance to guide vaccine strain selection.

## 1 Introduction

Seasonal influenza continues to pose a significant public health challenge worldwide, leading to annual outbreaks that cause considerable illness and death. Both influenza A and B viruses are responsible for seasonal epidemics. While influenza A viruses are primarily associated with pandemics and dominate most seasons, influenza B viruses contribute substantially to the seasonal disease burden and are particularly linked to severe illness in children and adolescents [1]. Influenza B viruses are divided into two antigenically distinct lineages: B/Victoria and B/Yamagata. Since the late 1980s, these lineages have co‐circulated globally, with varying patterns of dominance depending on region and year [2, 3, 4, 5]. Notably, B/Yamagata lineage viruses have not been detected in global surveillance since March 2020, raising questions regarding their ongoing circulation or potential extinction [6, 7, 8].

Vaccination is the primary strategy for influenza prevention. The World Health Organization (WHO) reviews global influenza surveillance data to update vaccine composition recommendations twice a year—for the Northern (NH) and Southern Hemispheres (SH) [9]. Previously, influenza vaccines were trivalent, containing one strain each of A(H1N1), A(H3N2), and B virus. However, the co‐circulation of both B lineages prompted the adoption of quadrivalent vaccines (QIVs) that include representatives from both lineages, B/Victoria and B/Yamagata, beginning with the 2013 SH season [10]. Due to the recent lack of B/Yamagata detections, a return to trivalent vaccines, excluding B/Yamagata, starting with the 2024 SH season, a decision that has also been continued in subsequent vaccine recommendations [11].

Despite the importance of influenza B, comprehensive molecular epidemiological studies remain limited, particularly in underrepresented regions such as countries with tropical climates. We have been conducting long‐term influenza surveillance studies in both Japan and Myanmar, which offer distinct epidemiological contexts [12, 13, 14, 15, 16]. Japan, a temperate country, experiences seasonal influenza peaks during the winter months and adopts the WHO vaccine recommendations for the NH. In contrast, Myanmar, located in the tropics, sees increased influenza activity primarily during the rainy season. Although geographically situated in the NH, Myanmar follows the WHO vaccine recommendations for the SH due to the seasonal timing of its epidemics [17]. To enable multiyear genomic comparisons, we focused on Japan and Myanmar, the two settings in our surveillance network with consecutive sample availability and full‐genome sequencing data. These countries represent markedly contrasting epidemiological contexts: Japan (temperate climate, winter influenza peaks) and Myanmar (tropical climate, rainy season peaks) are separated by approximately 5000 km and belong to different WHO regions (Western Pacific and Southeast Asia, respectively). This design allowed us to examine whether influenza B lineage transitions, clade emergence, and reassortment patterns showed similar dynamics despite differences in geography, climate, WHO region, and epidemic timing.

In this study, we analyzed influenza B viruses collected before the COVID‐19 pandemic, between 2016 and 2020 in Japan and Myanmar using whole‐genome sequencing. We aimed to describe seasonal trends, lineage dynamics, the emergence of reassortants, antiviral resistance mutations, and genetic differences between circulating strains in the two countries and the WHO‐recommended vaccine strains.

## 2 Material and Methods

### 2.1 Sample Collection

Influenza B viruses used in this study were collected through our previous surveillance activities in Japan and Myanmar [12, 13, 14, 15, 16]. In Japan, from January 2016 to March 2020, patients presenting with influenza‐like illness (ILI), including fever and respiratory symptoms, who sought medical attention at collaborating hospitals in 13 regions (Chiba, Gunma, Hokkaido, Kagawa, Kumamoto, Kyoto, Nagasaki, Nara, Niigata, Okinawa, Shizuoka, Tokyo, and Yamaguchi; see Figure S1 for locations) were enrolled in the study. Nasopharyngeal swab specimens were collected from these patients. Written informed consent was obtained from all patients or their guardians. Two swabs were collected from each patient: one was used at the collection site for rapid influenza screening using the Quick Navi kit (Denka Co., Ltd., Tokyo, Japan), and the other was placed in transport medium and transported domestically to Niigata University, where it was stored at −80°C until further virological analysis.

In Myanmar, from June 2016 to October 2019, patients visiting collaborating hospitals in three regions (Mandalay, Nay Pyi Taw, and Yangon; see Figure S1 for locations) were enrolled using the same protocol. Samples were stored at −80°C and transported to the testing facility twice a year via World Courier.

This study was approved by the Niigata University School of Medicine Ethical Committee (1178, 2015‐2533, 2018‐0317) and the Institutional Review Board, Ministry of Health and Sports, Myanmar (016516).

### 2.2 RNA Extraction

Total RNA was extracted from 140 μL of influenza‐positive clinical specimens, as well as from samples containing the culture supernatant and cells with isolated viruses, using the QIAamp Viral RNA Mini Kit (Qiagen, Hilden, Germany), according to the manufacturer's instructions.

### 2.3 Cycling Probe Real‐Time PCR for Lineage Determination

Complementary DNA (cDNA) was synthesized from the extracted RNA using universal primers (Uni‐11 and Uni‐12) and reverse transcription reagents, as previously reported [18, 19]. The resulting cDNA was subjected to cycling probe real‐time PCR to determine the lineage. This assay, established in our department, is designed to identify influenza B lineages based on the HA gene [15, 20]. Two fluorescent‐labeled chimeric RNA–DNA probes, labeled with 6‐carboxyfluorescein (FAM) and 6‐carboxy‐X‐rhodamine (ROX), were, respectively, designed to detect the target DNA sequences of influenza B/Victoria and B/Yamagata lineages. RT‐PCR was performed using 1 μL of sample cDNA under the following cycling conditions: initial denaturation at 95°C for 10 s, followed by denaturation at 95°C for 5 s, primer annealing at 57°C for 10 s, and fluorescence detection during extension at 72°C for 15 s. A total of 45 cycles was conducted. A cycle threshold (Ct) value of less than 38 was considered positive.

### 2.4 Viral Isolation

Virus isolation was carried out using the same procedures outlined in previous studies conducted by our research group [16]. Briefly, Madin–Darby Canine Kidney (MDCK) cells or MDCK‐SIAT1 cells were cultured in 48‐well plates at 37°C with 5% CO_2_. On the following day, clinical specimens were inoculated onto the cells and cultured at 34°C with 5% CO_2_. After inoculation, the cytopathic effect was monitored for 3 to 10 days. After cultivation, the culture supernatant and cells were stored at −80°C. Since 2018, MDCK‐SIAT1 cells have been used in place of MDCK cells. These cells, which express human 2,6‐sialyltransferase at high levels, show improved isolation rates for influenza viruses compared to conventional MDCK cells [21].

### 2.5 PCR Amplification for Next‐Generation Sequencing (NGS)

To amplify the whole genome of influenza B viruses, RNA extracted from isolated viruses was subjected to one‐step reverse transcription PCR to generate amplicons. Multiplex PCR was performed using the SuperScript III One‐Step RT‐PCR System with Platinum Taq (Invitrogen, Waltham, MA, USA). A previously established set of 13 primer cocktails was used [22], with primers targeting the PA gene applied at double concentration to enhance the amplification efficiency of internal genes. The thermal cycling conditions were as follows: reverse transcription at 45°C for 60 min and 55°C for 30 min; initial denaturation/enzyme activation at 94°C for 2 min; 5 cycles of 94°C for 20 s, 45°C for 30 s, and 68°C for 3 min; followed by 30 cycles of 94°C for 30 s, 57°C for 30 s, and 68°C for 3 min. Finally, the reaction was held at 4°C. The quality of PCR products was evaluated using the Agilent 4150 TapeStation (Agilent Technologies, Waldbronn, Germany) with the D5000 ScreenTape assay.

### 2.6 Library Preparation and Sequence Runs for NGS

Libraries for NGS were prepared using the QIAseq FX DNA Library CDI Kit (QIAGEN, Hilden, Germany). Amplified products were enzymatically fragmented into approximately 250‐bp fragments. Platform‐specific adapters were ligated to both ends of the DNA fragments. The resulting libraries were purified using the Agencourt AMPure XP Kit (Beckman Coulter, Indianapolis, IN, USA). The concentration of each purified library was measured using a Qubit Flex Fluorometer (Thermo Fisher Scientific, Waltham, MA, USA) with the Qubit 1X dsDNA HS Assay Kit. Sequencing was performed using the iSeq 100 System (Illumina, San Diego, CA, USA), and sequencing reads were obtained in FASTQ format.

### 2.7 NGS Data Analysis

Sequencing results were assembled and aligned using CLC Genomics Workbench software v.20.0.2 (QIAGEN, Hilden, Germany). The reference sequences used were B/Washington/02/2019 (B/Victoria lineage) and B/Phuket/3073/2013 (B/Yamagata lineage), which were the WHO‐recommended vaccine strains for the 2020 SH [23] and 2020–2021 NH influenza seasons [24]. The generated gene sequences were deposited in the public database Global Initiative on Sharing All Influenza Data (GISAID) (www.gisaid.org) (Table S4).

### 2.8 Phylogenetic Analysis

Phylogenetic trees were constructed for each segment using the sequences obtained through NGS. As references, additional sequences listed in Table S3 were included. These reference sequences cover WHO‐recommended vaccine strains selected during the study period as well as those recommended for the following season. Phylogenetic inference was conducted using the maximum‐likelihood method with 1000 bootstrap replicates. For each segment, the substitution model with the lowest corrected Akaike Information Criterion (AICc) was selected. All analyses were performed using MEGA version 7.0.26.

Clade identification based on the HA segment amino acid sequences was performed according to WHO classifications and also annotated on the phylogenetic trees of the other segments. To detect reassortment, we examined whether each virus isolate clustered within the same lineage or clade as in the HA segment on the phylogenetic trees constructed for the remaining segments.

### 2.9 Evaluation of Genetic Match With Vaccine Strains Based on HA Amino Acid Sequences

The HA sequences obtained from collected strains were compared with the WHO‐recommended vaccine strains corresponding to the time of sample collection to evaluate the genetic match, including antigenic sites. The reference sequences used for comparison are listed in Table S3.

## 3 Results

### 3.1 Sample Selection

**FIGURE 1:**
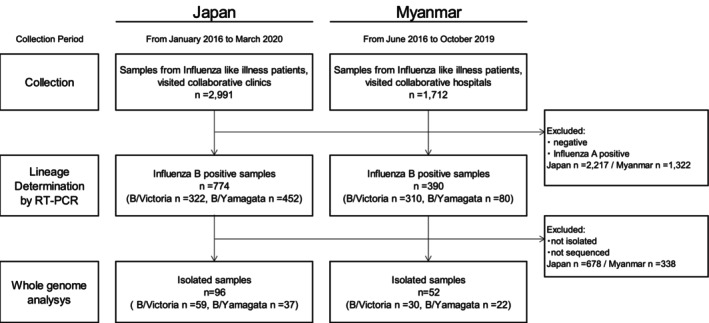
Flowchart for analysis sample selection. *Note:* Nasopharyngeal samples were collected from patients with influenza‐like illness who tested positive for influenza using rapid diagnostic kits at hospitals in both countries. Influenza B virus lineage identification was conducted using real‐time PCR on clinical or isolated samples. The samples were subjected to isolation culture using MDCK or MDCK‐SIAT1 cells, and sequencing was conducted using the isolated strains, resulting in a total of 148 whole‐genome sequences. Abbreviations: MDCK, Madin–Darby Canine Kidney; RT‐PCR, real‐time polymerase chain reaction.

During the study period from January 2016 to March 2020, a total of 4703 clinical specimens were collected, 2991 from Japan and 1712 from Myanmar (Figure 1). Of the 4703 clinical specimens, 1164 tested positive for influenza B by RT‐PCR and underwent lineage determination. Among these, 632 were classified as B/Victoria lineage (322 from Japan and 310 from Myanmar), and 532 as B/Yamagata lineage (452 from Japan and 80 from Myanmar). Whole‐genome sequencing was successfully performed on 148 influenza B virus isolates. Among the sequenced viruses, 89 were B/Victoria lineage (59 from Japan and 30 from Myanmar), and 59 were B/Yamagata lineage (37 from Japan and 22 from Myanmar). The number and lineage distribution of sequenced isolates by season (Japan) or calendar year (Myanmar) are presented in Tables S1 and S2. Isolates were selected to provide representative temporal and geographic coverage, with approximately 14–24 per season in Japan and 7–16 per year in Myanmar.

### 3.2 Epidemiological Trends of Influenza B Virus in Japan and Myanmar (2016–2020)

**FIGURE 2:**
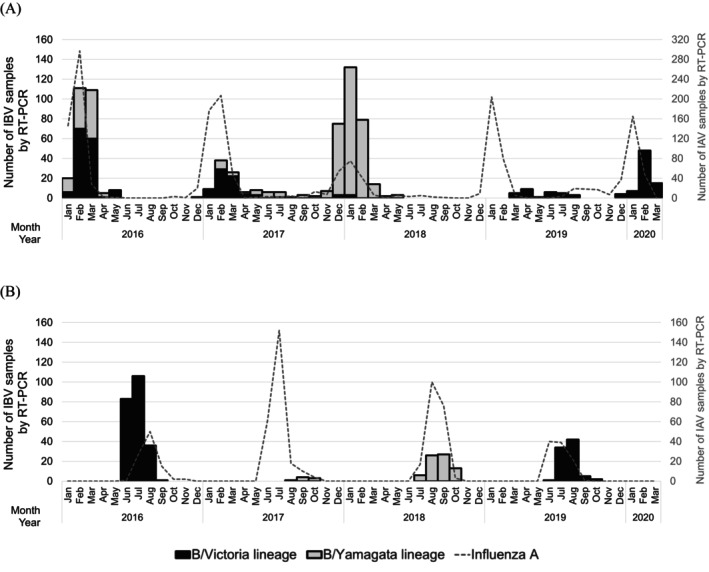
Epidemiologic trend of Influenza B Virus in Japan and Myanmar during the study period. *Note:* Epidemic curves based on RT‐PCR‐detected data from Japan (A) and Myanmar (B). Black bars represent the monthly detection count of B/Victoria lineage, while gray bars indicate B/Yamagata lineage. The number of analyzed samples is presented in Table 1. The dashed line represents the number of detected influenza A viruses. Abbreviations: IAV, influenza A virus; IBV, influenza B virus; RT‐PCR, real‐time polymerase chain reaction.

According to our RT‐PCR–based surveillance data shown in Figure 2A, Japan exhibited clear seasonal peaks in influenza B virus activity between December and March during most seasons, typically following the peak of influenza A activity. In 2017, Okinawa showed prolonged influenza B activity through September to October and appeared to transition directly into the subsequent season, indicating a regional deviation from the national trend (Figure S2). An exception was the 2018–2019 season, when no major influenza B epidemic was observed in Japan, despite concurrent influenza A circulation (Figure 2A); instead, low‐level B virus detections were reported from March to August 2019, again predominantly in Okinawa (Figure S2).

In contrast, influenza B activity in Myanmar was primarily observed during the rainy season, typically from June to October (Figure 2B). Distinct peaks of influenza B were recorded in 2016, 2018, and 2019, with the highest activity in mid‐year months. In 2017, detection levels were minimal. Influenza A activity generally preceded or overlapped with B virus circulation, with its peak occurring slightly earlier or concurrently in the same season. This tropical seasonal pattern contrasts with the wintertime influenza peaks consistently observed in Japan.

Over the course of the study, both countries demonstrated clear temporal shifts in lineage predominance (Tables 1 and 2 and Figure 2). In Japan, during the 2015–2016 season, both lineages co‐circulated, with B/Victoria accounting for 56.9% and B/Yamagata for 43.1% of detections. B/Victoria remained dominant in 2016–2017 (66.3%), but a major shift occurred in the 2017–2018 season, when B/Yamagata viruses became overwhelmingly predominant (98.1%). From the 2018–2019 season onward, only B/Victoria viruses were detected, indicating a lineage replacement. The last detection of B/Yamagata in Japan was in May 2018 in this study.

**TABLE 1:** Number of influenza B lineages detected in Japan during each season.

Season	Total	B/Victoria lineage (%)	B/Yamagata lineage (%)
2015–2016	253	144 (56.9%)	109 (43.1%)
2016–2017	104	69 (66.3%)	35 (33.7%)
2017–2018	314	6 (1.9%)	308 (98.1%)
2018–2019	29	29 (100%)	0 (0%)
2019–2020	74	74 (100%)	0 (0%)
**Total**	**774**	**322**	**452**

*Note:* A season refers to the period from October of the previous year to September of the following year. Only one case of mixed detection of the B/Victoria and B/Yamagata lineages was observed in the 2016–2017 season, and it was counted twice.

**TABLE 2:** Number of influenza B lineages detected in Myanmar during each year.

Year	Total	B/Victoria lineage (%)	B/Yamagata lineage (%)
2016	226	226 (100%)	0 (0%)
2017	8	0 (0%)	8 (100%)
2018	72	0 (0%)	72 (100%)
2019	84	84 (100%)	0 (0%)
2020	0	0 (–)	0 (–)
**Total**	**390**	**310**	**80**

*Note:* In 2020, no detections were made due to the study period ending before the typical influenza season.

In Myanmar, only one lineage was detected per year. In 2016, all detected influenza B viruses (*n* = 226) belonged to the B/Victoria lineage (Tables 1 and 2 and Figure 2). This shifted dramatically in 2017 and 2018, when all detections (*n* = 8 and *n* = 72, respectively) were B/Yamagata. A reversion to B/Victoria occurred in 2019, with 100% of detections belonging to this lineage. No influenza B viruses were detected in 2020, as the study was stopped before the typical influenza season due to the onset of the COVID‐19 pandemic. The last B/Yamagata detection in Myanmar occurred in October 2018.

### 3.3 Comprehensive Genome Analysis Reveals Clade Dynamics and Reassortment

**FIGURE 3:**
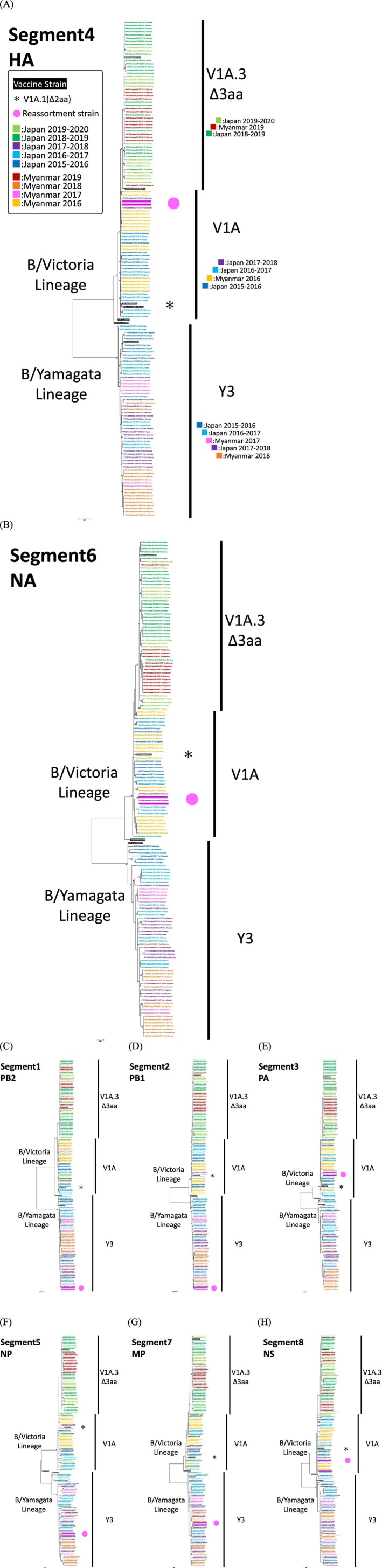
Maximum‐likelihood phylogenetic tree of eight segments of influenza B viruses circulating in Japan and Myanmar. *Note:* Comparison of sequences of 148 strains isolated in Japan and Myanmar between 2016 and 2020 with WHO‐recommended vaccine strains during the period. (A) HA, (B) NA, (C) PB2, (D) PB1, (E) PA, (F) NP, (G) MP, and (H) NS segments. The trees are color‐coded based on seasons and locations. The clades are based on the analysis results of the HA segment. Δ3aa indicates a 3‐amino acid deletion in HA. WHO‐recommended vaccine strains up to the next study period are highlighted in black. Asterisks (*) indicate vaccine strains with a 2‐amino acid deletion in HA (clade V1A.1). Strains that belonged to different lineages in other segments are highlighted in pink and marked with pink circles. The phylogenetic tree was inferred using the maximum‐likelihood method with 1000 bootstrap replicates, implemented in MEGA v.7.0.26. Branch values > 70% are indicated at the nodes. Abbreviations: HA, hemagglutinin; MP, matrix protein; NA, neuraminidase; NP, nucleocapsid protein; NS, nonstructural protein; PA, polymerase acid; PB1, polymerase basic 1; PB2, polymerase basic 2; WHO, world health organization.

Maximum‐likelihood phylogenetic trees were constructed for all eight genome segments (Figure 3), including HA (Segment 4), NA (Segment 6), and the six internal genes (PB2, PB1, PA, NP, MP, and NS). Based on HA sequences, B/Victoria viruses clustered into clades V1A and V1A.3, with V1A.3 characterized by a three–amino acid deletion (Δ162–164) (Figure 3A). V1A viruses were detected from 2016 to early 2018, while V1A.3 viruses emerged in Japan in 2019 and were also detected in Myanmar in the same year. Meanwhile, V1A.1 viruses with a two–amino acid deletion (Δ162–163) in B/Victoria lineage were not detected from Japan and Myanmar in this study. B/Brisbane/60/2008, a vaccine component for B/Victoria during 2015–2016 to 2017–2018 NH, and 2016 to 2018 SH, belonged to V1A in the HA phylogeny. B/Colorado/06/2017, a vaccine component selected for B/Victoria with a two–amino acid deletion (Δ162–163) in V1A.1 during 2018–2019 and 2019–2020 NH and 2019 SH, marked with asterisks (*) in Figure 3 consistently clustered in V1A clade but separate from V1A.3 viruses. To note, B/Maryland/15/2016, antigenically similar to B/Colorado/06/2017, was selected as a vaccine component in Japan during 2018–2019 and 2019–2020. In contrast, all B/Yamagata viruses clustered within clade Y3. The selected vaccine component for B/Yamagata, B/Phuket/3073/2013, from 2015–2016 to 2020–2021 NH and from 2016 to 2020 SH, fell into Y3 clade. For the remaining seven segments—PB2, PB1, PA, NP, NA, MP, and NS, B/Victoria lineage sequences clustered into clade V1A during 2016–2018 and into clade V1A.3 during 2019–2020, while B/Yamagata sequences consistently grouped within clade Y3. These clade classifications were consistent across segments, except in the two reassortant strains.

**FIGURE 4:**
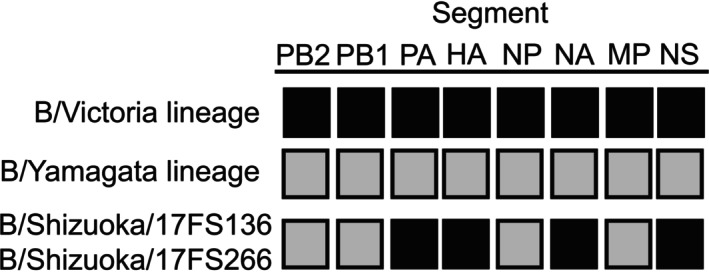
Reassortant influenza B strains identified in this study. *Note:* Black boxes represent gene segments derived from the B/Victoria lineage, while gray boxes indicate those from the B/Yamagata lineage. The two reassortant strains identified in this study were determined by whole‐genome phylogenetic analysis to possess four segments, including HA and NA, that were classified as belonging to the B/Victoria lineage, while the remaining segments were derived from the B/Yamagata lineage (see the phylogenetic trees in Figure 3). Abbreviations: HA, hemagglutinin; MP, matrix protein; NA, neuraminidase; NP, nucleocapsid protein; NS, nonstructural protein; PA, polymerase acid; PB1, polymerase basic 1; PB2, polymerase basic 2.

Two reassortant strains identified in Japan in 2018, B/Shizuoka/17FS136/2018 and B/Shizuoka/17FS266/2018, were detected through comparative phylogenetic analysis across all eight genome segments. The samples were collected 2 weeks apart, on January 16 and January 30, 2018, from patients at the same outpatient facility in the same city; however, no direct epidemiological link was confirmed between the two cases. These viruses harbored PA, HA, NA, and NS genes from the B/Victoria lineage, while PB2, PB1, NP, and MP genes belonged to the B/Yamagata lineage (Figure 4). The two reassortant strains exhibited a whole‐genome nucleotide identity of 99.97% (four nucleotide differences out of 13,748 bases), indicating close genetic relatedness. The reassortant genome of B/Shizuoka/17FS136 was confirmed by sequencing directly from a clinical specimen as well as from the isolate (Table S4). Sequencing data from the clinical specimen of B/Shizuoka/17FS266 were not available due to technical failure.

Among all sequenced strains, we screened the neuraminidase (NA) gene for amino acid substitutions associated with resistance to neuraminidase inhibitors (NAIs), and the polymerase acidic (PA) gene for substitutions associated with reduced susceptibility to cap‐dependent endonuclease (CEN) inhibitors. One Myanmar virus (B/Myanmar/16M003) harbored the E105K mutation in the NA gene, previously reported to confer reduced susceptibility to NAIs [25, 26]. However, no phenotypic susceptibility test against NAIs was conducted in this study. No mutations associated with reduced susceptibility to CEN were identified in the PA gene.

All sequences obtained in this study were submitted to GISAID (accession numbers 19062391‐19122602).

### 3.4 Amino Acid Differences in Hemagglutinin Gene Between Circulating and Vaccine Strains

The HA amino acid sequences of influenza B viruses collected in Japan and Myanmar were compared with WHO‐recommended vaccine strains corresponding to the respective influenza seasons (Tables 3 and 4).

**TABLE 3:** Summary of common mutation differences in the hemagglutinin amino acid sequences of B/Victoria lineage viruses analyzed in this study, compared with WHO‐recommended vaccine strains.

Strain		Clade	Match	Amino acid substitutions							
				120 loop					160 loop	120 loop	
				117	128	129	133	136	162–164	180	498
B/Brisbane/60/2008 (2015–2016 to 2017–2018 NH vaccine) (2016 to 2018 SH vaccine)		V1A		I		N					
Japan	2015–2016 (*n* = 9)	V1A	Yes	V		D					
	2016–2017 (*n* = 9)	V1A	Yes	V		D					
	2017–2018 (*n* = 4)	V1A	Yes	V		D					
Myanmar	2016 (*n* = 16)	V1A	Yes	V		D/G					
B/Colorado/06/2017 (2018–2019 and 2019–2020 NH vaccine) (2019 SH vaccine)		V1A.1			E	G	G	K	‐ ‐ D	V	K
Japan	2018–2019 (*n* = 23)	V1A.3	No		・	D	R	E	‐ ‐ —	I	R
	2019–2020 (*n* = 14)	V1A.3	No		K	D	R	E	‐ ‐ —	I	R
Myanmar	2019 (*n* = 14)	V1A.3	No		・	D	R	E	‐ ‐ —	I	R

*Note:* Gray‐shaded columns represent the vaccine strains recommended for each corresponding season. “Match” indicates whether the virus is genetically matched to the vaccine strain. Mutations located in antigenic sites (120‐ and 160‐loop) are indicated accordingly. A dot (·) represents an identical amino acid to the vaccine strain (no mutation), and a dash (–) denotes a deletion. Further details are provided in Tables S5 and S6.

**TABLE 4:** Summary of common mutation differences in the hemagglutinin amino acid sequences of B/Yamagata lineage viruses analyzed in this study, compared with WHO‐recommended vaccine strains.

Strain		Clade	Match	Amino acid substitutions	
				172	251
B/Phuket/3073/2013 (2015–2016 to 2020–2021 NH vaccine) (2016 to 2020 SH vaccine)		Y3		L	M
Japan	2015–2016 (*n* = 5)	Y3	Yes	Q	V/I
	2016–2017 (*n* = 15)	Y3	Yes	Q	V
	2017–2018 (*n* = 17)	Y3	Yes	Q	V
Myanmar	2017 (*n* = 7)	Y3	Yes	Q	V
	2018 (*n* = 5)	Y3	Yes	Q	V

*Note:* Gray‐shaded columns represent the vaccine strains that were consistently recommended throughout the study period. “Match” indicates whether the virus is genetically matched to the vaccine strain. Mutations located in antigenic sites were not found in this study. Further details are provided in Tables S7 and S8.

Among B/Victoria lineage viruses, strains isolated in Japan during the 2015–2016 to 2017–2018 seasons were classified as clade V1A, consistent with B/Brisbane/60/2008, the WHO‐recommended vaccine strain for those seasons. These viruses were genetically matched to the vaccine strain, though two substitutions—I117V and N129D—were consistently observed in the antigenic 120‐loop. Myanmar strains detected in 2016 also belonged to clade V1A and carried the same I117V and N129D substitutions.

In the 2018–2019 and 2019–2020 seasons, Japanese viruses shifted to clade V1A.3, while the vaccine strain B/Colorado/06/2017 belonged to clade V1A.1, indicating a clade mismatch. V1A.3 viruses showed a three–amino acid deletion (Δ162–164) in the 160‐loop, whereas the vaccine strain had only a two–amino acid deletion (Δ162–163). Additional 120‐loop mutations—G129D, G133R, K136E, and V180I—were also observed in V1A.3 strains detected during the 2018–2019 and 2019–2020 seasons. An E128K substitution, absent in earlier strains or vaccine components, was uniquely detected in Japanese viruses in the 2019–2020 season. Myanmar viruses from 2019 also belonged to clade V1A.3 and carried the same amino acid changes—Δ162–164 in the 160‐loop and G129D, G133R, K136E, and V180I in the 120‐loop—supporting a shared evolutionary path and further confirming a mismatch with the vaccine strain in Myanmar.

To resolve the mismatch between circulating strains and the vaccine strain, B/Washington/02/2019 (clade V1A.3), containing the three–amino acid deletion, was adopted by WHO as the updated vaccine component for the 2020 SH and 2020–2021 NH seasons.

For the B/Yamagata lineage, all viruses collected in Japan during the 2015–2016 to 2017–2018 seasons and in Myanmar during 2017 and 2018 were classified as clade Y3, consistent with the WHO‐recommended strain B/Phuket/3073/2013. In 2017, B/Yamagata lineage viruses were detected in Myanmar, whereas the trivalent vaccine used in the SH at that time contained only a B/Victoria lineage strain, B/Brisbane/60/2008‐like, showing a lineage mismatch. Both Japanese and Myanmar strains exhibited L172Q and M251V substitutions in the HA protein, which are located outside known antigenic regions.

Additionally, various sporadic amino acid substitutions were observed in the HA sequences of both Japanese and Myanmar strains when compared to the respective vaccine strains. These mutations are not considered antigenically significant and are therefore not individually described (Tables S5–S8).

## 4 Discussion

We conducted a comprehensive analysis of the molecular epidemiology of influenza B viruses circulating in Japan and Myanmar from 2016 to 2020, using whole‐genome sequencing. Our comparison of the two countries, separated by approximately 5000 km with markedly different climates and influenza seasonality, yielded several insights into influenza B virus evolution in Asia that would not have been evident from single‐country surveillance alone. The synchronized lineage transitions observed in both countries—B/Victoria in 2016, B/Yamagata dominance in 2017–2018, and reversion to B/Victoria in 2019—occurred despite different epidemic timing (winter peaks vs. rainy season peaks), suggesting that these shifts were driven by global circulation patterns rather than local factors. Similarly, the V1A.3 clade emerged in both countries during 2019, and the vaccine mismatch patterns were comparable despite different hemisphere vaccine recommendations (NH vs. SH formulations for Japan and Myanmar, respectively). The detection of inter‐lineage reassortants with B/Yamagata internal genes during the transition period in Japan demonstrates the complexity of lineage replacement mechanisms. Most notably, the parallel disappearance of B/Yamagata in both tropical (Myanmar, October 2018) and temperate (Japan, May 2018) settings several months before the COVID‐19 pandemic provides important evidence that the decline began independently of pandemic‐related disruptions. While broad epidemiological extrapolation from two countries is limited, these observations demonstrate that certain evolutionary dynamics—including lineage replacement, clade emergence, and vaccine mismatch patterns—transcend local seasonality and geographical separation, likely reflecting coordinated global or regional circulation of influenza B viruses in Asia.

According to our dataset, the last detections of B/Yamagata lineage viruses occurred in May 2018 in Japan and October 2018 in Myanmar. Globally, B/Yamagata viruses have not been genetically confirmed since March 2020, when the final verified case, B/Arizona/12/2020, was identified [27]. B/Yamagata emerged in 1983 after diverging from B/Victoria; since then, the two lineages have generally co‐circulated, although B/Victoria largely disappeared from surveillance in the 1990s and re‐emerged globally in the early 2000s [28]. During the 2000s and 2010s, B/Yamagata viruses frequently predominated in seasonal epidemics, especially among older adults, and were known for accumulating fewer amino acid changes in hemagglutinin (HA) compared to B/Victoria viruses, contributing to their slower antigenic drift [6]. Notably, the decline of the B/Yamagata lineage appears to have begun before the COVID‐19 pandemic. In our study, B/Yamagata viruses were last detected in 2018 in both Japan (May) and Myanmar (October), and global surveillance data likewise show reduced detections in the prepandemic period [6, 7, 8]. Despite intensified genomic surveillance during the COVID‐19 pandemic, no genetically confirmed B/Yamagata detections have been reported globally since early 2020 [6, 7, 8]. These findings suggest that the pandemic likely accelerated this decline through public health interventions that markedly altered influenza transmission dynamics [6, 8]. At the same time, the reduction in detections prior to the pandemic indicates that additional factors may have contributed to the apparent disappearance of the lineage, underscoring the importance of continued molecular surveillance to detect any potential resurgence. While sporadic detections have been reported in regions such as Kenya, Greece, and Spain in 2021 and 2022, these were either associated with live‐attenuated vaccines or lacked full‐genome confirmation [8]. In response to this apparent disappearance, the WHO Influenza Vaccine Composition Advisory Committee concluded in 2023 that inclusion of a B/Yamagata component in quadrivalent influenza vaccines is no longer warranted [29]. Consequently, many countries, including those in the European Union and Australia, have reverted to trivalent vaccine formulations containing only a B/Victoria component [30, 31]. Nevertheless, given that the B/Victoria lineage re‐emerged globally in the early 2000s after nearly a decade of undetectable circulation, the potential for B/Yamagata to reappear cannot be excluded. Continued global molecular surveillance remains essential to detect any resurgence early and to support evidence‐based vaccine strain updates.

In our study, B/Victoria lineage viruses carrying a three–amino acid deletion in the hemagglutinin (HA) gene (Δ162–164), classified as clade V1A.3, were detected in both Japan and Myanmar in 2019, whereas no viruses with a two–amino acid deletion were observed. Since 2016, the B/Victoria lineage has undergone notable genetic diversification, driven by the emergence of HA deletion variants that altered antigenic properties. Viruses with a two–amino acid deletion at HA positions 162–163 (clade 1A.1) first appeared between June 2016 and January 2017 and rapidly spread, particularly across North America, Europe, and Africa [32, 33]. These variants frequently carried additional mutations, such as D129G and I180V in the HA head and K371Q and V401I in the neuraminidase gene, suggesting compensatory adaptations to maintain viral fitness. Soon after, lineages with a three–amino acid deletion at positions 162–164, clades 1A.2, 1A.3, and 1A.4, emerged independently between late 2016 and 2017 [32]. Among these, clade 1A.3 became predominant across East and Southeast Asia by 2019 [34, 35]. In Thailand, similar to our findings in Myanmar, V1A.3 viruses were detected in 2019, indicating regional spread in Southeast Asia [36]. In Japan, Kato‐Miyashita et al. documented co‐circulation of two–amino acid deletion variants during the 2017–2018 and 2018–2019 seasons, alongside the emergence of three–deletion variants in 2018–2019 [34]. Although two–deletion variants were not identified in our Japanese dataset, the emergence of three–deletion viruses was consistent with these findings. Collectively, these observations suggest that V1A.3 variants became regionally dominant around 2019, replacing earlier clades such as V1A and V1A.1. Globally, by late 2019, three–deletion variants had become predominant in most regions, with exceptions in areas such as Madagascar, Mozambique, and parts of Central and South America, where two–deletion variants remained dominant [23]. These global trends highlight the rapid and widespread replacement of earlier B/Victoria variants by the more antigenically divergent V1A.3 clade.

Throughout the study period, we identified two instances of potential mismatch between circulating influenza B viruses and the vaccine strains recommended for the corresponding seasons. The first occurred in 2017, when only B/Yamagata lineage viruses were detected in Myanmar, yet the WHO‐recommended trivalent vaccine for the SH that year included only a B/Victoria lineage strain (B/Brisbane/60/2008), resulting in a lineage mismatch. Previous studies have demonstrated that vaccine effectiveness is substantially reduced when the vaccine contains a different B lineage than the circulating virus [37, 38]. In contrast, Japan had already adopted the quadrivalent influenza vaccine (QIV) containing both B/Victoria and B/Yamagata lineage antigens starting from the 2015–2016 season [39], which may have contributed to broader protection against influenza B viruses. The second mismatch involved antigenic differences within the B/Victoria lineage. During the 2018–2019 and 2019–2020 seasons in Japan, and in 2019 in Myanmar, the circulating B/Victoria viruses belonged to clade 1A.3, which harbors a three–amino acid deletion in the HA (Δ162–164). However, the vaccine strain used in those seasons, B/Colorado/06/2017, belonged to clade 1A.1 and carried only a two–amino acid deletion (Δ162–163). Although we did not perform hemagglutination inhibition (HI) assays, several studies have reported antigenic differences between these clades. According to the WHO, B/Victoria viruses with the three–amino acid deletion were poorly inhibited by ferret antisera raised against B/Colorado/06/2017‐like viruses, suggesting reduced cross‐reactivity [23]. Similar findings were reported by Kato‐Miyashita et al. [34] using ferret antisera and by Xie et al. [40] in studies using sera from vaccinated humans. The impact of this antigenic mismatch on vaccine effectiveness remains mixed: some studies reported moderate effectiveness despite the mismatch [41], while others found little to no protective effect [42]. To mitigate this mismatch, WHO selected B/Washington/02/2019 (V1A.3; Δ162–164) as the B/Victoria component for the 2020 SH and the 2020–2021 NH seasons. Given that influenza vaccine production requires over 6 months, vaccine strain selection must be finalized well before the start of the flu season [43]. Therefore, timely and accurate molecular surveillance remains essential for informing vaccine updates and improving protection against evolving influenza B viruses.

Notably, we identified two reassortant influenza B viruses in samples collected in Japan. These viruses were detected 2 weeks apart from patients who visited the same hospital. The two strains exhibited a whole‐genome nucleotide identity of 99.97% (four nucleotide differences out of 13,748 bases), indicating close genetic relatedness. This high degree of similarity, along with the shared genomic constellation and close collection dates, suggests that the result of independent reassortment events but rather part of a locally circulating subpopulation. Although genetic reassortment in influenza B has been previously reported [44, 45, 46, 47, 48], to our knowledge, the specific segment constellation identified in this study, PA, HA, NA, and NS segments derived from the B/Victoria lineage, and PB2, PB1, NP, and MP segments derived from the B/Yamagata lineage, has not been documented in the literature to date. Previous studies suggest that reassortants retaining homologous combinations of PB1, PB2, and HA may have a replication advantage [49, 50]. In contrast, the reassortants identified in our study lacked such a configuration, which may explain their limited transmission and apparent confinement to a single region. Nevertheless, these findings underscore the potential for reassortment during periods of lineage co‐circulation and highlight the importance of whole‐genome sequencing in detecting rare reassortant variants that might otherwise go undetected by partial gene analyses.

The selection of Japan and Myanmar for this comparative genomic analysis was based on several methodological considerations. First, these were the only two locations in our surveillance network with continuous, multi‐year sample collection and complete whole‐genome sequencing data for all eight segments, enabling comprehensive reassortment analysis. Second, the approximately 5000 km geographical separation and contrasting epidemiological contexts (temperate winter peaks vs. tropical rainy season peaks) allowed us to test whether evolutionary patterns were locally driven or reflected broader regional/global dynamics. Third, although the countries belong to different WHO regions (Western Pacific for Japan and Southeast Asia for Myanmar), they follow different hemisphere vaccine recommendations, providing a natural experiment to examine vaccine mismatch patterns. The synchronized lineage transitions, clade emergence, and parallel B/Yamagata disappearance observed despite these differences suggest that influenza B evolution in Asia is coordinated across diverse climatic and geographic settings. While this two‐country comparison cannot establish continent‐wide patterns, it provides important evidence that certain evolutionary dynamics operate at scales larger than individual countries, supporting the value of multicountry genomic surveillance in understanding regional influenza B circulation across Asia.

This study has several limitations. First, in Myanmar, samples were collected from only three geographic regions, which may not provide a complete picture of nationwide influenza B virus circulation. Although surveillance in Japan covered a broader area, from Hokkaido to Okinawa, it may still have been insufficient to fully capture nationwide trends and regional variability. Second, this study did not include HI testing. While HI remains a reference method for antigenic characterization, practical constraints limit its use in large molecular epidemiologic studies, including altered RBC agglutination in recent B viruses [51], labor‐intensive ferret antisera production with interlaboratory variability [52, 53], and operator‐dependent variation [54]. Conversely, sequence‐based surveillance, now central to WHO guidance [55], captures the major antigenically relevant HA1 substitutions that explain much antigenic drift [34]. Our whole‐genome approach enables comprehensive segment monitoring, reproducible data sharing, and retrospective analysis. We incorporated published antigenic findings on V1A and V1A.3 strains, including HA deletions (Δ162–164). While functional serology would provide complementary information, our sequence‐based approach provides a robust framework for interpreting antigenic drift and vaccine‐strain match. Third, this study did not assess clinical outcomes or vaccine effectiveness across different lineages or clades, which limits our ability to draw conclusions about the real‐world implications of the observed genetic differences. Despite these limitations, the mutation patterns identified were consistent with those reported in WHO vaccine strain selection updates, supporting the relevance and robustness of our genomic findings. Fourth, our phylogenetic analysis was restricted to strains from Japan and Myanmar, which were the only locations in our surveillance network with sufficient continuous multi‐year whole‐genome data to enable the comprehensive eight‐segment reassortment analysis that was central to this study. While incorporating sequences from additional Asian countries could provide broader regional context, such expansion would necessitate phylogeographic modeling to properly characterize spatial–temporal transmission dynamics—an analytical approach that would substantially alter the scope and objectives of this genomic surveillance study. Despite this geographic limitation, the contrasting epidemiological contexts of Japan and Myanmar (separated by ~5000 km, different climates, different WHO regions, and different influenza seasonality) enabled us to identify synchronized evolutionary patterns that suggest coordinated regional circulation, as discussed above. Future studies incorporating broader Asian sequences with phylogeographic approaches would provide valuable complementary insights into regional transmission networks and lineage spread across the continent.

## 5 Conclusions

This study provides valuable insights into the evolutionary dynamics of influenza B viruses in Asia and demonstrates the utility of whole‐genome surveillance in understanding lineage transitions, reassortment events, and vaccine mismatch. As global vaccine policy shifts in response to the apparent disappearance of B/Yamagata lineage viruses, continued molecular surveillance will be essential to inform vaccine composition and guide effective public health responses.

## Author Contributions

**Yusuke Ichikawa:** conceptualization; methodology; investigation; data curation; writing – original draft preparation; visualization. **Reiko Saito:** conceptualization; resources; writing – original draft preparation; writing – review and editing; supervision; project administration; funding acquisition. **Irina Chon:** conceptualization; methodology; software; validation; investigation. **Wint Wint Phyu:** investigation; data curation. **Yadanar Kyaw:** investigation; supervision. **Su Mon Kyaw Win:** investigation. **Japanese Influenza Collaborative Study Group:** investigation. **Sayaka Yoshioka:** investigation. **Yuyang Sun:** investigation. **Jiaming Li:** investigation. **Tri Bayu Purnama:** conceptualization. **Keita Wagatsuma:** conceptualization; writing – review and editing. **Tsutomu Tamura:** conceptualization; data curation. **Hisami Watanabe:** conceptualization; writing – review and editing; supervision; project administration; funding acquisition. **Moe Myat Aye:** investigation. **Swe Setk:** investigation; supervision. **Htay Htay Tin:** investigation; supervision. All authors have read and agreed to the published version of the manuscript.

## Funding

This study is supported by the Japan Initiative for Global Research Network on Infectious Diseases (J‐GRID) from Japan Agency for Medical Research and Development (AMED) (15fm0108009h0001–19fm0108009h0005 and 20wm0125005h0001–24wm0125005h0005), the Health Labour Sciences Research Grant by the Ministry of Health, Labour and Welfare, and Sciences, Japan (21HA2003), and Shionogi & Co. Ltd.

## Conflicts of Interest

Yusuke Ichikawa is an employee of Denka Co. Ltd. All other authors report no conflicts of interest.

## Supporting information


**Table S1:** Number of influenza B viruses from Japan for which whole‐genome sequences were obtained in this study.
**Table S2:** Number of influenza B viruses from Myanmar for which whole‐genome sequences were obtained in this study.
**Table S3:** List of reference influenza B virus strains and their GISAID accession numbers used for phylogenetic analysis and vaccine strain comparison.
**Table S5:** List of amino acid mutations in the hemagglutinin sequences of B/Victoria lineage viruses analyzed in this study from Japan, compared with WHO‐recommended vaccine strains.
**Table S6:** List of amino acid mutations in the hemagglutinin sequences of B/Victoria lineage viruses analyzed in this study from Myanmar, compared with WHO‐recommended vaccine strains.
**Table S7:** List of amino acid mutations in the hemagglutinin sequences of B/Yamagata lineage viruses analyzed in this study from Japan, compared with WHO‐recommended vaccine strains.
**Table S8:** List of amino acid mutations in the hemagglutinin sequences of B/Yamagata lineage viruses analyzed in this study from Myanmar, compared with WHO‐recommended vaccine strains.
**Figure S1:** Geographical distribution of influenza viruses in this study.
**Figure S2:** Epidemiologic trend of influenza B virus in Japan during the study period.
**Figure S3:** Epidemiologic trend of influenza B virus in Japan and Myanmar during the study period based on WHO data.

**Table S4:** List of strain names and GISAID accession numbers for all eight segments of influenza B viruses sequenced in this study.

## Data Availability

All whole‐genome sequence data generated in this study have been deposited in GISAID. The accession numbers are listed in Table S4. Other data that support the findings of this study are not available for public access.
