# Supplementary material for: Molecular Epidemiology and Genetic Diversity of Influenza B Viruses Based on Whole‐Genome Analysis in Japan and Myanmar, 2016–2020
Source: Influenza Other Respir Viruses. 2026 Feb 9;20(2):e70234. doi: 10.1111/irv.70234 (PMC12886744; doi:10.1111/irv.70234)
Supplement: Supplementary file 1 — Table S1: Number of influenza B viruses from Japan for which whole‐genome sequences were obtained in this study. Table S2: Number of influenza B viruses from Myanmar for which whole‐genome sequences were obtained in this study. Table S3: List of reference influenza B virus strains and their GISAID accession numbers used for phylogenetic analysis and vaccine strain comparison. Table S5: List of amino acid mutations in the hemagglutinin sequences of B/Victoria lineage viruses analyzed in this study from Japan, compared with WHO‐recommended vaccine strains. Table S6: List of amino acid mutations in the hemagglutinin sequences of B/Victoria lineage viruses analyzed in this study from Myanmar, compared with WHO‐recommended vaccine strains. Table S7: List of amino acid mutations in the hemagglutinin sequences of B/Yamagata lineage viruses analyzed in this study from Japan, compared with WHO‐recommended vaccine strains. Table S8: List of amino acid mutations in the hemagglutinin sequences of B/Yamagata lineage viruses analyzed in this study from Myanmar, compared with WHO‐recommended vaccine strains. Figure S1: Geographical distribution of influenza viruses in this study. Figure S2: Epidemiologic trend of influenza B virus in Japan during the study period. Figure S3: Epidemiologic trend of influenza B virus in Japan and Myanmar during the study period based on WHO data. [file IRV-20-e70234-s002.docx]

**Supplementary Table 1. Number of influenza B viruses from Japan for which whole-genome sequences were obtained in this study.**

| **Season** | **Total (%)** | **B/Victoria lineage (%)** | **B/Yamagata lineage (%)** |
| --- | --- | --- | --- |
| 2015–2016 | 14 (5.5%) | 9 (6.3%) | 5 (4.6%) |
| 2016–2017 | 24 (23.1%) | 9 (13.0%) | 15 (42.8%) |
| 2017–2018 | 21 (6.6%) | 4 (66.7%) | 17 (5.5%) |
| 2018–2019 | 23 (79.3%) | 23 (79.3%) | 0 ( – ) |
| 2019–2020 | 14 (18.9%) | 14 (18.9%) | 0 ( – ) |
| **Total** | **96 (12.4%)** | **59 (18.3%)** | **37 (8.1%)** |

Notes: A season refers to the period from October of the previous year to September of the following year. The percentages represent the proportion of lineage-determined strains shown in Table 1.

**Supplementary Table 2. Number of influenza B viruses from Myanmar for which whole-genome sequences were obtained in this study.**

| **Year** | **Total (%)** | **B/Victoria lineage (%)** | **B/Yamagata lineage (%)** |
| --- | --- | --- | --- |
| 2016 | 16 (7.1%) | 16 (7.1%) | 0 ( – ) |
| 2017 | 7 (87.5%) | 0 ( – ) | 7 (87.5%) |
| 2018 | 15 (20.8%) | 0 ( – ) | 15 (20.8%) |
| 2019 | 14 (16.7%) | 14 (16.7%) | 0 ( – ) |
| 2020 | 0 ( – ) | 0 ( – ) | 0 ( – ) |
| **Total** | **52 (13.3%)** | **30 (9.7%)** | **22 (27.5%)** |

Notes: The percentages represent the proportion of lineage-determined strains shown in Table 2. In 2020, no detections were made due to the study period ending before the typical influenza season.

**Supplementary Table 3. List of reference influenza B virus strains and their GISAID accession numbers used for phylogenetic analysis and vaccine strain comparison.**

| Lineage | Strain | Remarks | ID | PB2 | PB1 | PA | HA | NP | NA | MP | NS |
| --- | --- | --- | --- | --- | --- | --- | --- | --- | --- | --- | --- |
| B/Victoria | B/Brisbane/60/2008 | From 2015–2016 to 2017–2018 NH Vaccine strain  From 2016 to 2018 SH Vaccine strain | EPI_ISL_129017 | EPI  2021111 | EPI  2021112 | EPI  2021110 | EPI  394895 | EPI  2021108 | EPI  394894 | EPI  2021109 | EPI  394893 |
|  | B/Texas/2/2013 | From 2015–2016 to 2017–2018 Vaccine strain in Japan | EPI_ISL_394869 | － | － | － | EPI  1605916 | － | － | － | － |
|  | B/Colorado/06/2017 | 2018–2019 and 2019–2020 NH Vaccine strain  2019 SH Vaccine strain | EPI_ISL_277231 | EPI  1269610 | EPI  1269609 | EPI  1269608 | EPI  1056637 | EPI  1056633 | EPI  1056636 | EPI  1056635 | EPI  1056634 |
|  | B/Maryland/15/2016(BX-69A) | 2018–2019 and 2019–2020 Vaccine strain in Japan | EPI_ISL_278409 | － | － | － | EPI1061863 | － | － | － | － |
|  | B/Washington/02/2019 | 2020–2021 NH Vaccine strain  2020 SH Vaccine strain | EPI_ISL_353725 | EPI  1430133 | EPI  1430134 | EPI  1482038 | EPI  1430136 | EPI  1430130 | EPI  1430135 | EPI  1430132 | EPI  1430131 |
|  | B/Victoria/705/2018(BVR-11) | 2020–2021 Vaccine strain in Japan | EPI_ISL_397113 | － | － | － | EPI  1618362 | － | － | － | － |
| B/Yamagata | B/Phuket/3073/2013 | From 2015–2016 to 2020–2021 NH Vaccine strain  From 2016 to 2020 SH Vaccine strain  From 2015–2016 to 2020–2021 Vaccine strain in Japan | EPI_ISL_166958 | EPI  1269607 | EPI  552546 | EPI  544265 | EPI  544267 | EPI  626300 | EPI  544266 | EPI  552545 | EPI  547695 |

Notes: This table includes the strains used for phylogenetic analysis and hemagglutinin amino acid sequence comparison with vaccine strains in this study. It covers WHO-recommended vaccine strains selected during the study period as well as those recommended for the subsequent season. For Japan, WHO-recommended strains for the Northern Hemisphere were considered under the assumption of QIV, while for Myanmar, Southern Hemisphere recommendations were referenced under the assumption of TIV. Additionally, for Japan, the CVVs selected as the manufacturing strain were included.

Abbreviations: PB2, polymerase basic 2; PB1, polymerase basic 1; PA, polymerase acid; HA, hemagglutinin; NP, nucleocapsid protein; NA, neuraminidase; MP, matrix protein; NS, nonstructural protein; WHO, world health organization; QIV, quadrivalent influenza vaccines; TIV, trivalent influenza vaccines; CVVs, candidate vaccine viruses.

**Supplementary Table 5. List of amino acid mutations in the hemagglutinin sequences of B/Victoria lineage viruses analyzed in this study** **from Japan, compared with WHO-recommended vaccine strains.**

| Strain | Clade | Amino Acid Substitutions | | | | | | | | | | | | | | | | | |
| --- | --- | --- | --- | --- | --- | --- | --- | --- | --- | --- | --- | --- | --- | --- | --- | --- | --- | --- | --- |
|  |  | 47 | 117^†^ | 129^†^ | 136^†^ | 146^†^ | 155 | 156 | 177^†^ | 353 |  | | | | | | | | |
| B/Brisbane/60/2008 | V1A | T | **I** | **N** | K | I | T | M | V | I |  |  |  |  |  |  |  |  |  |
| B/Texas/2/2013^‡^ | V1A | ・ | **・** | **D** | ・ | V | ・ | ・ | ・ | ・ |  |  |  |  |  |  |  |  |  |
| 2015–2016 Japan Viruses (n =9) | V1A | ・ | **V** | **D** | E | ・ | ・ | V | ・ | V |  |  |  |  |  |  |  |  |  |
| 2016–2017 Japan Viruses (n =9) | V1A | I | **V** | **D** | ・ | ・ | A | ・ | I | ・ |  |  |  |  |  |  |  |  |  |
| 2017–2018 Japan Viruses (n =4) | V1A | ・ | **V** | **D** | ・ | ・ | ・ | ・ | ・ | ・ |  |  |  |  |  |  |  |  |  |
|  | | 110 | 127^†^ | 128^†^ | 129^†^ | 133^†^ | 136^†^ | 162-164^†^ | 180^†^ | 189 | 199^†^ | 252 | 341 | 395 | 478 | 498 | 526 | 547 | 548 |
| B/Colorado/06/2017 | V1A.1 | L | A | **E** | **G** | **G** | **K** | **--D** | V | T | T | V | A | I | V | **K** | D | V | T |
| B/Maryland/15/2016(BX-69A) ^‡^ | V1A.1 | ・ | ・ | **・** | **D** | **・** | **・** | **--D** | ・ | ・ | I | ・ | ・ | ・ | ・ | **・** | ・ | ・ | ・ |
| 2018–2019 Japan Viruses (n =23) | V1A.3 | I | T | **・** | **D** | **R** | **E** | **---** | I | S | ・ | R | T | ・ | ・ | **R** | E | ・ | A |
| 2019–2020 Japan Viruses (n =14) | V1A.3 | ・ | T | **K** | **D** | **R** | **E** | **---** | I | ・ | ・ | ・ | E | V | I | **R** | ・ | I | A |
|  | | 68 | 110 | 127^†^ | 128^†^ | 133^†^ | 189 | 199^†^ | 252 | 341 | 395 | 478 | 526 | 547 | 548 | 563 |  | | |
| B/Washington/02/2019^§^ | V1A.3 | G | L | A | **E** | R | T | T | V | A | I | V | D | V | T | N |  |  |  |
| B/Victoria/705/2018(BVR-11)^‡^ | V1A.3 | S | ・ | ・ | **・** | G | ・ | I | ・ | ・ | ・ | ・ | ・ | ・ | ・ | S |  |  |  |
| 2018–2019 Japan Viruses (n =23) | V1A.3 | ・ | I | T | **・** | ・ | S | ・ | R | T | ・ | ・ | E | ・ | A | ・ |  |  |  |
| 2019–2020 Japan Viruses (n=14) | V1A.3 | ・ | ・ | T | **K** | ・ | ・ | ・ | ・ | E | V | I | ・ | I | A | ・ |  |  |  |

Notes: Gray-shaded columns indicate the vaccine strains recommended for each respective influenza season. Mutations commonly observed among the analyzed strains are shown in bold. A dagger (^†^) denotes antigenic sites. A double dagger (^‡^) indicate candidate vaccine viruses that were actually used as manufacturing strains for vaccines in Japan. A section sign (^§^) indicate the vaccine strains recommended for the subsequent season within the study period. A dot (·) represents an amino acid identical to that of the corresponding vaccine strain (i.e., no mutation), and a dash (–) indicates a deletion.

**Supplementary Table 6. List of amino acid mutations in the hemagglutinin sequences of B/Victoria lineage viruses analyzed in this study** **from Myanmar, compared with WHO-recommended vaccine strains.**

| Strain | Clade | Amino Acid Substitutions | | | | | | | | | |
| --- | --- | --- | --- | --- | --- | --- | --- | --- | --- | --- | --- |
|  |  | 81 | 90 | 117^†^ | 121^†^ | 129^†^ | 221 | 341 | 415 |  | |
| B/Brisbane/60/2008 | V1A | V | V | **I** | T | **N** | T | A | G |  |  |
| 2016 Myanmar Viruses (n =16) | V1A | A | I | **V** | I | **D**/G | I | S | S |  |  |
|  | | 118^†^ | 121^†^ | 129^†^ | 133^†^ | 136^†^ | 162-164^†^ | 180^†^ | 254 | 415 | 498 |
| B/Colorado/06/2017 | V1A.1 | R | T | **G** | **G** | **K** | **--D** | **V** | K | G | **K** |
| 2019 Myanmar Viruses (n =14) | V1A.3 | K | I | **D** | **R** | **E** | **---** | **I** | R | S | **R** |
|  | | 118^†^ | 121^†^ | 254 | 415 |  | | | | | |
| B/Washington/02/2019^§^ | V1A.3 | R | T | K | G |  |  |  |  |  |  |
| 2019 Myanmar Viruses (n =14) | V1A.3 | K | I | R | S |  |  |  |  |  |  |

Notes: Gray-shaded columns indicate the vaccine strains recommended for each respective influenza season. Mutations commonly observed among the analyzed strains are shown in bold. A dagger (^†^) denotes antigenic sites. A section sign (^§^) indicate the vaccine strains recommended for the subsequent season within the study period. A dash (–) indicates a deletion.

**Supplementary Table 7. List of amino acid mutations in the hemagglutinin sequences of B/Yamagata lineage viruses analyzed in this study** **from Japan, compared with WHO-recommended vaccine strains.**

| Strain | Clade | Amino Acid Substitutions | | | | | | | | | | | | | | | | |
| --- | --- | --- | --- | --- | --- | --- | --- | --- | --- | --- | --- | --- | --- | --- | --- | --- | --- | --- |
|  |  | 19 | 48^†^ | 73 | 76 | 121^†^ | 123^†^ | 150^†^ | 172 | 211 | 217 | 251 | 253 | 255 | 256 | 278 | 312 | 540 |
| B/Phuket/3073/2013^‡^ | Y3 | A | R | V | T | T | N | I | **L** | K | N | **M** | K | G | K | R | K | A |
| 2015–2016 Japan Viruses (n =5) | Y3 | ・ | ・ | ・ | ・ | ・ | ・ | ・ | **Q** | R | ・ | **V/I** | ・ | E | ・ | ・ | ・ | ・ |
| 2016–2017 Japan Viruses (n =15) | Y3 | V | K | M | I | ・ | K | ・ | **Q** | ・ | S | **V** | ・ | ・ | ・ | ・ | R | V |
| 2017–2018 Japan Viruses (n =17) | Y3 | ・ | ・ | ・ | ・ | I | ・ | N | **Q** | ・ | ・ | **V** | T/R | ・ | R | K | ・ | ・ |

Notes: Gray-shaded columns indicate the vaccine strains recommended for each respective influenza season. Mutations commonly observed among the analyzed strains are shown in bold. A dagger (^†^) denotes antigenic sites. A double dagger (^‡^) indicate candidate vaccine viruses that were actually used as manufacturing strains for vaccines in Japan. A dot (·) represents an amino acid identical to that of the corresponding vaccine strain (i.e., no mutation).

**Supplementary Table 8. List of amino acid mutations in the hemagglutinin sequences of B/Yamagata lineage viruses analyzed in this study** **from Myanmar, compared with WHO-recommended vaccine strains.**

| HA amino acid sequences | clade | Amino Acid Substitutions | | | | | | | | | |
| --- | --- | --- | --- | --- | --- | --- | --- | --- | --- | --- | --- |
|  |  | 123^†^ | 124^†^ | 164^†^ | 172 | 181^†^ | 188 | 250 | 251 | 505 | 558 |
| B/Phuket/3073/2013 | Y3 | N | V | N | **L** | T | T | M | **M** | A | V |
| 2017 Myanmar Viruses (n =7) | Y3 | ・ | ・ | S | **Q** | ・ | A | ・ | **V** | ・ | ・ |
| 2018 Myanmar Viruses (n =15) | Y3 | K | A | ・ | **Q** | A | ・ | L | **V** | V | I |

Notes: Gray-shaded columns indicate the vaccine strains recommended for each respective influenza season. Mutations commonly observed among the analyzed strains are shown in bold. A dagger (^†^) denotes antigenic sites. A dot (·) represents an amino acid identical to that of the corresponding vaccine strain (i.e., no mutation).

1. (B)

**Supplementary Figure 1. Geographical distribution of influenza viruses in this study.**

Notes: Locations where samples were collected: Myanmar (A) and Japan (B). In Japan, samples were obtained from regions spanning from Hokkaido in the north to Okinawa in the south.

(A)

(B)

**
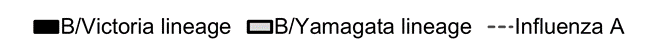
**

**Supplementary Figure 2. Epidemiologic trend of influenza B virus in Japan during the study period.**

Notes: Epidemic curves based on RT-PCR-detected data from Okinawa (A) and other regions of Japan (B). These are shown separately to highlight regional differences in the timing of virus detection, as Okinawa exhibited distinct circulation periods compared to the rest of the country. Black bars represent the monthly detection count of B/Victoria lineage, while gray bars indicate B/Yamagata lineage.

Abbreviations: RT-PCR, real-time polymerase chain reaction; IBV: influenza B virus.

(A)

(B)

**
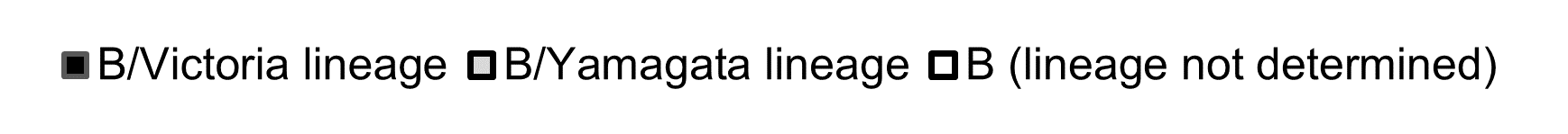
**

**Supplementary Figure 3. Epidemiologic trend of influenza B virus in Japan and Myanmar during the study period based on WHO data.**

Notes: Epidemic curves based on the number of reported specimens from Japan (A) and Myanmar (B). Data source: FluNet (https://www.who.int/tools/flunet). Black bars represent the monthly detection counts of B/Victoria lineage viruses, gray bars indicate B/Yamagata lineage viruses, and white bars denote cases where the lineage was not determined.
