# Supplementary material for: Molecular Epidemiology and Genetic Diversity of Influenza B Viruses Based on Whole‐Genome Analysis in Japan and Myanmar, 2016–2020
Source: Influenza Other Respir Viruses. 2026 Feb 9;20(2):e70234. doi: 10.1111/irv.70234 (PMC12886744; doi:10.1111/irv.70234)
Supplement: Supplementary file 2 — Table S4: List of strain names and GISAID accession numbers for all eight segments of influenza B viruses sequenced in this study. [file IRV-20-e70234-s001.docx]

**Supplementary Table 4. List of strain names and GISAID accession numbers for all eight segments of influenza B viruses sequenced in this study.**

| Lineage | Strain name | Collection Date  (Year/Month/Date) | ID | PB2 | PB1 | PA | HA | NP | NA | MP | NS |
| --- | --- | --- | --- | --- | --- | --- | --- | --- | --- | --- | --- |
| B/Victoria | B/Niigata/15F057/2016 isolated | 2016/1/25 | EPI_ISL_19062391 | EPI3221961 | EPI3226385 | EPI3226386 | EPI3226387 | EPI3226388 | EPI3226389 | EPI3226390 | EPI3226391 |
| B/Victoria | B/Niigata/15F326/2016 isolated | 2016/2/10 | EPI_ISL_19066085 | EPI3226741 | EPI3226742 | EPI3226743 | EPI3226745 | EPI3226747 | EPI3226748 | EPI3226751 | EPI3226753 |
| B/Victoria | B/Gunma/15G077/2016 isolated | 2016/3/8 | EPI_ISL_19071031 | EPI3230420 | EPI3230421 | EPI3230422 | EPI3230423 | EPI3230424 | EPI3230425 | EPI3230426 | EPI3230427 |
| B/Victoria | B/Gunma/15G090/2016 isolated | 2016/3/24 | EPI_ISL_19071032 | EPI3230428 | EPI3230429 | EPI3230430 | EPI3230431 | EPI3230432 | EPI3230433 | EPI3230434 | EPI3230435 |
| B/Victoria | B/Hokkaido/15H077/2016 isolated | 2016/2/15 | EPI_ISL_19071033 | EPI3230436 | EPI3230437 | EPI3230438 | EPI3230439 | EPI3230440 | EPI3230441 | EPI3230442 | EPI3230443 |
| B/Victoria | B/Hokkaido/15H097/2016 isolated | 2016/2/24 | EPI_ISL_19071059 | EPI3230551 | EPI3230552 | EPI3230553 | EPI3230554 | EPI3230555 | EPI3230556 | EPI3230557 | EPI3230558 |
| B/Victoria | B/Kyoto/15K053/2016 isolated | 2016/2/23 | EPI_ISL_19071061 | EPI3230559 | EPI3230560 | EPI3230561 | EPI3230562 | EPI3230563 | EPI3230564 | EPI3230565 | EPI3230566 |
| B/Victoria | B/Nagasaki/15N040/2016 isolated | 2016/2/16 | EPI_ISL_19071062 | EPI3230567 | EPI3230568 | EPI3230569 | EPI3230570 | EPI3230571 | EPI3230572 | EPI3230573 | EPI3230574 |
| B/Victoria | B/Nagasaki/15N048/2016 isolated | 2016/2/26 | EPI_ISL_19071063 | EPI3230575 | EPI3230576 | EPI3230577 | EPI3230578 | EPI3230579 | EPI3230580 | EPI3230581 | EPI3230582 |
| B/Victoria | B/Niigata/16F082/2017 isolated | 2017/1/23 | EPI_ISL_19071064 | EPI3230583 | EPI3230584 | EPI3230585 | EPI3230586 | EPI3230587 | EPI3230588 | EPI3230589 | EPI3230590 |
| B/Victoria | B/Niigata/16F379/2017 isolated | 2017/2/18 | EPI_ISL_19071065 | EPI3230591 | EPI3230592 | EPI3230593 | EPI3230594 | EPI3230595 | EPI3230596 | EPI3230597 | EPI3230598 |
| B/Victoria | B/Niigata/16F520/2017 isolated | 2017/3/10 | EPI_ISL_19071066 | EPI3230599 | EPI3230600 | EPI3230601 | EPI3230602 | EPI3230603 | EPI3230604 | EPI3230605 | EPI3230606 |
| B/Victoria | B/Nagasaki/16N005/2016 isolated | 2016/12/28 | EPI_ISL_19071067 | EPI3230607 | EPI3230608 | EPI3230609 | EPI3230610 | EPI3230611 | EPI3230612 | EPI3230613 | EPI3230614 |
| B/Victoria | B/Nagasaki/16N014/2017 isolated | 2017/1/28 | EPI_ISL_19071160 | EPI3230615 | EPI3230616 | EPI3230617 | EPI3230618 | EPI3230619 | EPI3230620 | EPI3230621 | EPI3230622 |
| B/Victoria | B/Nagasaki/16N028/2017 isolated | 2017/1/5 | EPI_ISL_19071161 | EPI3230623 | EPI3230624 | EPI3230625 | EPI3230626 | EPI3230627 | EPI3230628 | EPI3230629 | EPI3230630 |
| B/Victoria | B/Okinawa/16T016/2017 isolated | 2017/2/28 | EPI_ISL_19071162 | EPI3230631 | EPI3230632 | EPI3230633 | EPI3230634 | EPI3230635 | EPI3230636 | EPI3230637 | EPI3230638 |
| B/Victoria | B/Okinawa/16T022/2017 isolated | 2017/3/25 | EPI_ISL_19071163 | EPI3230639 | EPI3230640 | EPI3230641 | EPI3230642 | EPI3230643 | EPI3230644 | EPI3230645 | EPI3230646 |
| B/Victoria | B/Okinawa/16T050/2017 isolated | 2017/7/7 | EPI_ISL_19071164 | EPI3230647 | EPI3230648 | EPI3230649 | EPI3230650 | EPI3230651 | EPI3230652 | EPI3230653 | EPI3230654 |
| B/Victoria | B/Shizuoka/17FS023/2017 isolated | 2017/12/18 | EPI_ISL_19071175 | EPI3230655 | EPI3230656 | EPI3230657 | EPI3230658 | EPI3230659 | EPI3230660 | EPI3230661 | EPI3230662 |
| B/Victoria | B/Shizuoka/17FS041/2017 isolated | 2017/12/28 | EPI_ISL_19071176 | EPI3230663 | EPI3230664 | EPI3230665 | EPI3230666 | EPI3230667 | EPI3230668 | EPI3230669 | EPI3230670 |
| B/Victoria | B/Shizuoka/17FS136/2018 original | 2018/1/16 | EPI_ISL_19071177 | EPI3230671 | EPI3230672 | EPI3230673 | EPI3230674 | EPI3230675 | EPI3230676 | EPI3230677 | EPI3230678 |
| B/Victoria | B/Shizuoka/17FS136/2018 isolated | 2018/1/16 | EPI_ISL_19071178 | EPI3230679 | EPI3230680 | EPI3230681 | EPI3230682 | EPI3230683 | EPI3230684 | EPI3230685 | EPI3230686 |
| B/Victoria | B/Shizuoka/17FS266/2018 isolated | 2018/1/30 | EPI_ISL_19071179 | EPI3230687 | EPI3230688 | EPI3230689 | EPI3230690 | EPI3230691 | EPI3230692 | EPI3230693 | EPI3230694 |
| B/Victoria | B/Gunma/18FS323/2019 isolated | 2019/5/2 | EPI_ISL_19071180 | EPI3230695 | EPI3230696 | EPI3230697 | EPI3230698 | EPI3230699 | EPI3230700 | EPI3230701 | EPI3230702 |
| B/Victoria | B/Okinawa/18T030/2019 isolated | 2019/3/4 | EPI_ISL_19071181 | EPI3230703 | EPI3230704 | EPI3230705 | EPI3230706 | EPI3230707 | EPI3230708 | EPI3230709 | EPI3230710 |

Abbreviations: PB2, polymerase basic 2; PB1, polymerase basic 1; PA, polymerase acid; HA, hemagglutinin; NP, nucleocapsid protein; NA, neuraminidase; MP, matrix protein; NS, nonstructural protein.

**Supplementary Table 4. List of strain names and GISAID accession numbers for all eight segments of influenza B viruses sequenced in this study.**

| Lineage | Strain name | Collection Date  (Year/Month/Date) | ID | PB2 | PB1 | PA | HA | NP | NA | MP | NS |
| --- | --- | --- | --- | --- | --- | --- | --- | --- | --- | --- | --- |
| B/Victoria | B/Okinawa/18T031/2019 isolated | 2019/3/6 | EPI_ISL_19071282 | EPI3230711 | EPI3230712 | EPI3230713 | EPI3230714 | EPI3230715 | EPI3230716 | EPI3230717 | EPI3230718 |
| B/Victoria | B/Okinawa/18T032/2019 isolated | 2019/3/12 | EPI_ISL_19071283 | EPI3230719 | EPI3230720 | EPI3230721 | EPI3230722 | EPI3230723 | EPI3230724 | EPI3230725 | EPI3230726 |
| B/Victoria | B/Okinawa/18T033/2019 isolated | 2019/3/22 | EPI_ISL_19071284 | EPI3230727 | EPI3230728 | EPI3230729 | EPI3230730 | EPI3230731 | EPI3230732 | EPI3230733 | EPI3230734 |
| B/Victoria | B/Okinawa/18T034/2019 isolated | 2019/3/25 | EPI_ISL_19071285 | EPI3230735 | EPI3230736 | EPI3230737 | EPI3230738 | EPI3230739 | EPI3230740 | EPI3230741 | EPI3230742 |
| B/Victoria | B/Okinawa/18T037/2019 isolated | 2019/4/3 | EPI_ISL_19071286 | EPI3230743 | EPI3230744 | EPI3230745 | EPI3230746 | EPI3230747 | EPI3230748 | EPI3230749 | EPI3230750 |
| B/Victoria | B/Okinawa/18T039/2019 isolated | 2019/4/11 | EPI_ISL_19071287 | EPI3230751 | EPI3230752 | EPI3230753 | EPI3230754 | EPI3230755 | EPI3230756 | EPI3230757 | EPI3230758 |
| B/Victoria | B/Okinawa/18T040/2019 isolated | 2019/4/12 | EPI_ISL_19071303 | EPI3230759 | EPI3230760 | EPI3230761 | EPI3230762 | EPI3230763 | EPI3230764 | EPI3230765 | EPI3230766 |
| B/Victoria | B/Okinawa/18T041/2019 isolated | 2019/4/15 | EPI_ISL_19071375 | EPI3230767 | EPI3230768 | EPI3230769 | EPI3230770 | EPI3230771 | EPI3230772 | EPI3230773 | EPI3230774 |
| B/Victoria | B/Okinawa/18T042/2019 isolated | 2019/4/16 | EPI_ISL_19071376 | EPI3230775 | EPI3230776 | EPI3230777 | EPI3230778 | EPI3230779 | EPI3230780 | EPI3230781 | EPI3230782 |
| B/Victoria | B/Okinawa/18T043/2019 isolated | 2019/4/17 | EPI_ISL_19071449 | EPI3230783 | EPI3230784 | EPI3230785 | EPI3230786 | EPI3230787 | EPI3230788 | EPI3230789 | EPI3230790 |
| B/Victoria | B/Okinawa/18T044/2019 isolated | 2019/4/23 | EPI_ISL_19071579 | EPI3230791 | EPI3230792 | EPI3230793 | EPI3230794 | EPI3230795 | EPI3230796 | EPI3230797 | EPI3230798 |
| B/Victoria | B/Okinawa/18T047/2019 isolated | 2019/6/12 | EPI_ISL_19071580 | EPI3230799 | EPI3230800 | EPI3230801 | EPI3230802 | EPI3230803 | EPI3230804 | EPI3230805 | EPI3230806 |
| B/Victoria | B/Okinawa/18T049/2019 isolated | 2019/6/20 | EPI_ISL_19071581 | EPI3230807 | EPI3230808 | EPI3230809 | EPI3230810 | EPI3230811 | EPI3230816 | EPI3230812 | EPI3230814 |
| B/Victoria | B/Okinawa/18T050/2019 isolated | 2019/6/25 | EPI_ISL_19071585 | EPI3230819 | EPI3230820 | EPI3230821 | EPI3230822 | EPI3230824 | EPI3230826 | EPI3230827 | EPI3230828 |
| B/Victoria | B/Okinawa/18T051/2019 isolated | 2019/6/27 | EPI_ISL_19071590 | EPI3230833 | EPI3230834 | EPI3230835 | EPI3230836 | EPI3230838 | EPI3230839 | EPI3230841 | EPI3230842 |
| B/Victoria | B/Okinawa/18T052/2019 isolated | 2019/6/29 | EPI_ISL_19071594 | EPI3230848 | EPI3230850 | EPI3230851 | EPI3230852 | EPI3230853 | EPI3230854 | EPI3230855 | EPI3230856 |
| B/Victoria | B/Okinawa/18T055/2019 isolated | 2019/7/10 | EPI_ISL_19071597 | EPI3230862 | EPI3230864 | EPI3230865 | EPI3230866 | EPI3230867 | EPI3230869 | EPI3230870 | EPI3230871 |
| B/Victoria | B/Okinawa/18T057/2019 isolated | 2019/7/24 | EPI_ISL_19071599 | EPI3230874 | EPI3230875 | EPI3230876 | EPI3230877 | EPI3230878 | EPI3230879 | EPI3230880 | EPI3230882 |
| B/Victoria | B/Okinawa/18T061/2019 isolated | 2019/7/31 | EPI_ISL_19071604 | EPI3230896 | EPI3230897 | EPI3230900 | EPI3230901 | EPI3230902 | EPI3230904 | EPI3230905 | EPI3230907 |
| B/Victoria | B/Okinawa/18T063/2019 isolated | 2019/8/1 | EPI_ISL_19071607 | EPI3230922 | EPI3230923 | EPI3230925 | EPI3230926 | EPI3230929 | EPI3230931 | EPI3230932 | EPI3230933 |
| B/Victoria | B/Okinawa/18T084/2019 isolated | 2019/8/26 | EPI_ISL_19071615 | EPI3230945 | EPI3230946 | EPI3230947 | EPI3230949 | EPI3230950 | EPI3230953 | EPI3230955 | EPI3230957 |
| B/Victoria | B/Tokyo/19FS176/2020 isolated | 2020/2/3 | EPI_ISL_19084574 | EPI3250022 | EPI3250023 | EPI3250024 | EPI3250025 | EPI3250026 | EPI3250027 | EPI3250028 | EPI3250029 |
| B/Victoria | B/Tokyo/19FS178/2020 isolated | 2020/2/10 | EPI_ISL_19084575 | EPI3250030 | EPI3250031 | EPI3250032 | EPI3250033 | EPI3250034 | EPI3250035 | EPI3250036 | EPI3250037 |
| B/Victoria | B/Tokyo/19FS179/2020 isolated | 2020/2/12 | EPI_ISL_19084576 | EPI3250038 | EPI3250039 | EPI3250040 | EPI3250041 | EPI3250042 | EPI3250043 | EPI3250044 | EPI3250045 |
| B/Victoria | B/Kyoto/19FS219/2020 isolated | 2020/2/8 | EPI_ISL_19084577 | EPI3250046 | EPI3250047 | EPI3250048 | EPI3250049 | EPI3250050 | EPI3250051 | EPI3250052 | EPI3250053 |

Abbreviations: PB2, polymerase basic 2; PB1, polymerase basic 1; PA, polymerase acid; HA, hemagglutinin; NP, nucleocapsid protein; NA, neuraminidase; MP, matrix protein; NS, nonstructural protein.

**Supplementary Table 4. List of strain names and GISAID accession numbers for all eight segments of influenza B viruses sequenced in this study.**

| Lineage | Strain name | Collection Date  (Year/Month/Date) | ID | PB2 | PB1 | PA | HA | NP | NA | MP | NS |
| --- | --- | --- | --- | --- | --- | --- | --- | --- | --- | --- | --- |
| B/Victoria | B/Tokyo/19FS243/2020 isolated | 2020/3/3 | EPI_ISL_19084578 | EPI3250054 | EPI3250055 | EPI3250056 | EPI3250057 | EPI3250058 | EPI3250059 | EPI3250060 | EPI3250061 |
| B/Victoria | B/Hokkaido/19FS254/2020 isolated | 2020/1/31 | EPI_ISL_19084579 | EPI3250065 | EPI3250067 | EPI3250069 | EPI3250072 | EPI3250073 | EPI3250074 | EPI3250076 | EPI3250077 |
| B/Victoria | B/Hokkaido/19FS268/2020 isolated | 2020/2/12 | EPI_ISL_19084582 | EPI3250086 | EPI3250087 | EPI3250088 | EPI3250090 | EPI3250091 | EPI3250092 | EPI3250094 | EPI3250099 |
| B/Victoria | B/Hokkaido/19FS269/2020 isolated | 2020/2/13 | EPI_ISL_19084584 | EPI3250103 | EPI3250106 | EPI3250107 | EPI3250110 | EPI3250111 | EPI3250114 | EPI3250115 | EPI3250116 |
| B/Victoria | B/Hokkaido/19FS277/2020 isolated | 2020/2/19 | EPI_ISL_19084587 | EPI3250123 | EPI3250126 | EPI3250127 | EPI3250128 | EPI3250129 | EPI3250130 | EPI3250131 | EPI3250133 |
| B/Victoria | B/Kyoto/19FS281/2020 isolated | 2020/2/12 | EPI_ISL_19086696 | EPI3254315 | EPI3254316 | EPI3254317 | EPI3254318 | EPI3254319 | EPI3254320 | EPI3254321 | EPI3254322 |
| B/Victoria | B/Kyoto19FS285/2020 isolated | 2020/2/18 | EPI_ISL_19086697 | EPI3254323 | EPI3254324 | EPI3254325 | EPI3254326 | EPI3254327 | EPI3254328 | EPI3254329 | EPI3254330 |
| B/Victoria | B/Kyoto/19FS298/2020 isolated | 2020/3/7 | EPI_ISL_19086698 | EPI3254331 | EPI3254332 | EPI3254333 | EPI3254334 | EPI3254335 | EPI3254336 | EPI3254337 | EPI3254338 |
| B/Victoria | B/Okinawa/19T029/2019 isolated | 2019/12/3 | EPI_ISL_19086700 | EPI3254343 | EPI3254344 | EPI3254345 | EPI3254346 | EPI3254347 | EPI3254348 | EPI3254349 | EPI3254350 |
| B/Victoria | B/Okinawa/19T062/2020 isolated | 2020/2/13 | EPI_ISL_19086701 | EPI3254351 | EPI3254352 | EPI3254353 | EPI3254354 | EPI3254355 | EPI3254356 | EPI3254357 | EPI3254358 |
| B/Yamagata | B/Niigata/15F570/2016 isolated | 2016/3/23 | EPI_ISL_19086702 | EPI3254359 | EPI3254360 | EPI3254361 | EPI3254362 | EPI3254383 | EPI3254391 | EPI3254392 | EPI3254393 |
| B/Yamagata | B/Gunma/15G086/2016 isolated | 2016/3/17 | EPI_ISL_19086707 | EPI3254394 | EPI3254395 | EPI3254396 | EPI3254397 | EPI3254399 | EPI3254401 | EPI3254402 | EPI3254403 |
| B/Yamagata | B/Hokkaido/15H019/2016 isolated | 2016/2/1 | EPI_ISL_19086709 | EPI3254404 | EPI3254405 | EPI3254406 | EPI3254407 | EPI3254408 | EPI3254409 | EPI3254410 | EPI3254411 |
| B/Yamagata | B/Kyoto/15K003/2016 isolated | 2016/1/12 | EPI_ISL_19086710 | EPI3254412 | EPI3254413 | EPI3254414 | EPI3254415 | EPI3254416 | EPI3254417 | EPI3254418 | EPI3254419 |
| B/Yamagata | B/Nagasaki/15N055/2016 isolated | 2016/3/1 | EPI_ISL_19086711 | EPI3254420 | EPI3254421 | EPI3254422 | EPI3254423 | EPI3254424 | EPI3254425 | EPI3254426 | EPI3254427 |
| B/Yamagata | B/Niigata/16F284/2017 isolated | 2017/2/8 | EPI_ISL_19086712 | EPI3254428 | EPI3254429 | EPI3254430 | EPI3254431 | EPI3254432 | EPI3254433 | EPI3254434 | EPI3254435 |
| B/Yamagata | B/Niigata/16F377/2017 isolated | 2017/2/17 | EPI_ISL_19086713 | EPI3254436 | EPI3254437 | EPI3254438 | EPI3254439 | EPI3254440 | EPI3254441 | EPI3254442 | EPI3254443 |
| B/Yamagata | B/Niigata/16F541/2017 isolated | 2017/3/17 | EPI_ISL_19086714 | EPI3254444 | EPI3254445 | EPI3254446 | EPI3254447 | EPI3254448 | EPI3254449 | EPI3254450 | EPI3254451 |
| B/Yamagata | B/Shizuoka/16S091/2017 isolated | 2017/2/20 | EPI_ISL_19086715 | EPI3254452 | EPI3254453 | EPI3254454 | EPI3254455 | EPI3254456 | EPI3254457 | EPI3254458 | EPI3254459 |
| B/Yamagata | B/Okinawa/16T010/2017 isolated | 2017/2/4 | EPI_ISL_19086716 | EPI3254460 | EPI3254461 | EPI3254462 | EPI3254463 | EPI3254464 | EPI3254465 | EPI3254466 | EPI3254467 |
| B/Yamagata | B/Okinawa/16T013/2017 isolated | 2017/2/20 | EPI_ISL_19086717 | EPI3254468 | EPI3254469 | EPI3254470 | EPI3254471 | EPI3254472 | EPI3254473 | EPI3254474 | EPI3254475 |
| B/Yamagata | B/Okinawa/16T041/2017 isolated | 2017/6/5 | EPI_ISL_19086718 | EPI3254602 | EPI3254603 | EPI3254604 | EPI3254605 | EPI3254606 | EPI3254607 | EPI3254608 | EPI3254609 |
| B/Yamagata | B/Okinawa/16T045/2017 isolated | 2017/6/16 | EPI_ISL_19086736 | EPI3254610 | EPI3254758 | EPI3254896 | EPI3254897 | EPI3254898 | EPI3254899 | EPI3254900 | EPI3254901 |
| B/Yamagata | B/Okinawa/16T046/2017 isolated | 2017/6/20 | EPI_ISL_19086836 | EPI3254902 | EPI3254903 | EPI3254904 | EPI3254905 | EPI3254906 | EPI3254907 | EPI3254908 | EPI3254909 |
| B/Yamagata | B/Okinawa/16T047/2017 isolated | 2017/6/28 | EPI_ISL_19086839 | EPI3254910 | EPI3254911 | EPI3254912 | EPI3254913 | EPI3254914 | EPI3254915 | EPI3254916 | EPI3254917 |

Abbreviations: PB2, polymerase basic 2; PB1, polymerase basic 1; PA, polymerase acid; HA, hemagglutinin; NP, nucleocapsid protein; NA, neuraminidase; MP, matrix protein; NS, nonstructural protein.

**Supplementary Table 4. List of strain names and GISAID accession numbers for all eight segments of influenza B viruses sequenced in this study.**

| Lineage | Strain name | Collection Date  (Year/Month/Date) | ID | PB2 | PB1 | PA | HA | NP | NA | MP | NS |
| --- | --- | --- | --- | --- | --- | --- | --- | --- | --- | --- | --- |
| B/Yamagata | B/Okinawa/16T048/2017 isolated | 2017/7/5 | EPI_ISL_19086855 | EPI3254918 | EPI3254919 | EPI3254920 | EPI3254921 | EPI3254922 | EPI3254923 | EPI3254924 | EPI3254925 |
| B/Yamagata | B/Okinawa/16T049/2017 isolated | 2017/7/5 | EPI_ISL_19086856 | EPI3254926 | EPI3254927 | EPI3254928 | EPI3254929 | EPI3254930 | EPI3254933 | EPI3254931 | EPI3254932 |
| B/Yamagata | B/Okinawa/16T051/2017 isolated | 2017/7/10 | EPI_ISL_19086857 | EPI3254934 | EPI3254935 | EPI3254936 | EPI3254937 | EPI3254938 | EPI3254939 | EPI3254940 | EPI3254941 |
| B/Yamagata | B/Okinawa/16T052/2017 isolated | 2017/7/10 | EPI_ISL_19086858 | EPI3254942 | EPI3254943 | EPI3254944 | EPI3254945 | EPI3254946 | EPI3254947 | EPI3254948 | EPI3254949 |
| B/Yamagata | B/Okinawa/16T053/2017 isolated | 2017/7/12 | EPI_ISL_19086859 | EPI3254950 | EPI3254951 | EPI3254952 | EPI3254953 | EPI3254954 | EPI3254955 | EPI3254956 | EPI3254957 |
| B/Yamagata | B/Nagasaki/17FS004/2017 isolated | 2017/12/21 | EPI_ISL_19090212 | EPI3255620 | EPI3255622 | EPI3255623 | EPI3255624 | EPI3255625 | EPI3255628 | EPI3255630 | EPI3255632 |
| B/Yamagata | B/Kyoto/17FS015/2017 isolated | 2017/12/22 | EPI_ISL_19090215 | EPI3255642 | EPI3255643 | EPI3255644 | EPI3255645 | EPI3255646 | EPI3255647 | EPI3255648 | EPI3255649 |
| B/Yamagata | B/Shizuoka/17FS018/2017 isolated | 2017/11/27 | EPI_ISL_19090459 | EPI3255668 | EPI3255669 | EPI3255670 | EPI3255671 | EPI3255672 | EPI3255673 | EPI3255674 | EPI3255675 |
| B/Yamagata | B/Gunma/17FS037/2017 isolated | 2017/12/26 | EPI_ISL_19090516 | EPI3255676 | EPI3255677 | EPI3255678 | EPI3255679 | EPI3255680 | EPI3255681 | EPI3255682 | EPI3255683 |
| B/Yamagata | B/Shizuoka/17FS042/2018 isolated | 2018/1/4 | EPI_ISL_19090519 | EPI3255684 | EPI3255685 | EPI3255686 | EPI3255687 | EPI3255688 | EPI3255689 | EPI3255690 | EPI3255691 |
| B/Yamagata | B/Shizuoka/17FS047/2018 isolated | 2018/1/9 | EPI_ISL_19090594 | EPI3255692 | EPI3255693 | EPI3255694 | EPI3255695 | EPI3255696 | EPI3255697 | EPI3255698 | EPI3255692 |
| B/Yamagata | B/Nara/17FS053/2017 isolated | 2017/12/12 | EPI_ISL_19090595 | EPI3255700 | EPI3255701 | EPI3255702 | EPI3255703 | EPI3255704 | EPI3255705 | EPI3255706 | EPI3255707 |
| B/Yamagata | B/Hokkaido/17FS090/2017 isolated | 2017/12/5 | EPI_ISL_19090599 | EPI3255708 | EPI3255709 | EPI3255710 | EPI3255711 | EPI3255712 | EPI3255713 | EPI3255714 | EPI3255715 |
| B/Yamagata | B/Shizuoka/17FS133/2018 isolated | 2018/1/15 | EPI_ISL_19090702 | EPI3255716 | EPI3255717 | EPI3255718 | EPI3255735 | EPI3255763 | EPI3255791 | EPI3255814 | EPI3255840 |
| B/Yamagata | B/Shizuoka/17FS134/2018 isolated | 2018/1/15 | EPI_ISL_19090734 | EPI3255852 | EPI3255853 | EPI3255854 | EPI3255855 | EPI3255856 | EPI3255857 | EPI3255858 | EPI3255859 |
| B/Yamagata | B/Shizuoka/17FS138/2018 isolated | 2018/1/16 | EPI_ISL_19090741 | EPI3255860 | EPI3255861 | EPI3255862 | EPI3255863 | EPI3255864 | EPI3255865 | EPI3255866 | EPI3255867 |
| B/Yamagata | B/Shizuoka/17FS139/2018 isolated | 2018/1/16 | EPI_ISL_19090746 | EPI3255868 | EPI3255869 | EPI3255870 | EPI3255871 | EPI3255872 | EPI3255873 | EPI3255874 | EPI3255875 |
| B/Yamagata | B/Shizuoka/17FS141/2018 isolated | 2018/1/17 | EPI_ISL_19090751 | EPI3255876 | EPI3255877 | EPI3255878 | EPI3255879 | EPI3255880 | EPI3255881 | EPI3255882 | EPI3255883 |
| B/Yamagata | B/Shizuoka/17FS143/2018 isolated | 2018/1/17 | EPI_ISL_19090755 | EPI3255884 | EPI3255885 | EPI3255886 | EPI3255887 | EPI3255888 | EPI3255889 | EPI3255890 | EPI3255891 |
| B/Yamagata | B/Shizuoka/17FS188/2018 isolated | 2018/1/18 | EPI_ISL_19090770 | EPI3255892 | EPI3255893 | EPI3255894 | EPI3255895 | EPI3255896 | EPI3255897 | EPI3255898 | EPI3255899 |
| B/Yamagata | B/Shizuoka/17FS190/2018 isolated | 2018/1/19 | EPI_ISL_19090772 | EPI3255900 | EPI3255901 | EPI3255902 | EPI3255903 | EPI3255904 | EPI3255905 | EPI3255906 | EPI3255907 |
| B/Yamagata | B/Okinawa/17T073/2018 isolated | 2018/3/23 | EPI_ISL_19090775 | EPI3255908 | EPI3255909 | EPI3255910 | EPI3255911 | EPI3255912 | EPI3255913 | EPI3255914 | EPI3255915 |
| B/Victoria | B/Myanmar/16M003/2016 isolated | 2016/6/20 | EPI_ISL_19090779 | EPI3255916 | EPI3255917 | EPI3255918 | EPI3255919 | EPI3255920 | EPI3255921 | EPI3255922 | EPI3255923 |
| B/Victoria | B/Myanmar/16M005/2016 isolated | 2016/6/15 | EPI_ISL_19107783 | EPI3262462 | EPI3262463 | EPI3262464 | EPI3262465 | EPI3262466 | EPI3262467 | EPI3262468 | EPI3262469 |
| B/Victoria | B/Myanmar/16M018/2016 isolated | 2016/6/29 | EPI_ISL_19107865 | EPI3262470 | EPI3262471 | EPI3262472 | EPI3262473 | EPI3262474 | EPI3262475 | EPI3262476 | EPI3262477 |

Abbreviations: PB2, polymerase basic 2; PB1, polymerase basic 1; PA, polymerase acid; HA, hemagglutinin; NP, nucleocapsid protein; NA, neuraminidase; MP, matrix protein; NS, nonstructural protein.

**Supplementary Table 4. List of strain names and GISAID accession numbers for all eight segments of influenza B viruses sequenced in this study.**

| Lineage | Strain name | Collection Date  (Year/Month/Date) | ID | PB2 | PB1 | PA | HA | NP | NA | MP | NS |
| --- | --- | --- | --- | --- | --- | --- | --- | --- | --- | --- | --- |
| B/Victoria | B/Myanmar/16M074/2016 isolated | 2016/7/4 | EPI_ISL_19107866 | EPI3262478 | EPI3262479 | EPI3262480 | EPI3262481 | EPI3262482 | EPI3262483 | EPI3262484 | EPI3262485 |
| B/Victoria | B/Myanmar/16M124/2016 isolated | 2016/6/29 | EPI_ISL_19107867 | EPI3262486 | EPI3262487 | EPI3262488 | EPI3262489 | EPI3262490 | EPI3262491 | EPI3262492 | EPI3262493 |
| B/Victoria | B/Myanmar/16M160/2016 isolated | 2016/6/20 | EPI_ISL_19107868 | EPI3262494 | EPI3262495 | EPI3262496 | EPI3262497 | EPI3262498 | EPI3262499 | EPI3262500 | EPI3262501 |
| B/Victoria | B/Myanmar/16M189/2016 isolated | 2016/7/15 | EPI_ISL_19107869 | EPI3262502 | EPI3262503 | EPI3262504 | EPI3262505 | EPI3262506 | EPI3262507 | EPI3262508 | EPI3262509 |
| B/Victoria | B/Myanmar/16M250/2016 isolated | 2016/7/30 | EPI_ISL_19107870 | EPI3262510 | EPI3262511 | EPI3262512 | EPI3262513 | EPI3262514 | EPI3262515 | EPI3262516 | EPI3262517 |
| B/Victoria | B/Myanmar/16M254/2016 isolated | 2016/7/30 | EPI_ISL_19107871 | EPI3262518 | EPI3262519 | EPI3262520 | EPI3262521 | EPI3262522 | EPI3262525 | EPI3262523 | EPI3262524 |
| B/Victoria | B/Myanmar/16M281/2016 isolated | 2016/8/1 | EPI_ISL_19107872 | EPI3262526 | EPI3262527 | EPI3262528 | EPI3262529 | EPI3262530 | EPI3262531 | EPI3262532 | EPI3262533 |
| B/Victoria | B/Myanmar/16M363/2016 isolated | 2016/8/10 | EPI_ISL_19107873 | EPI3262534 | EPI3262535 | EPI3262536 | EPI3262537 | EPI3262538 | EPI3262539 | EPI3262540 | EPI3262541 |
| B/Victoria | B/Myanmar/16M385/2016 isolated | 2016/8/15 | EPI_ISL_19107960 | EPI3262875 | EPI3262876 | EPI3262877 | EPI3262878 | EPI3262879 | EPI3262906 | EPI3262880 | EPI3262881 |
| B/Victoria | B/Myanmar/16M444/2016 isolated | 2016/8/11 | EPI_ISL_19107964 | EPI3262907 | EPI3262908 | EPI3262909 | EPI3262910 | EPI3262911 | EPI3262912 | EPI3262914 | EPI3262915 |
| B/Victoria | B/Myanmar/16M487/2016 isolated | 2016/8/1 | EPI_ISL_19107965 | EPI3262916 | EPI3262917 | EPI3262918 | EPI3262919 | EPI3262920 | EPI3262921 | EPI3262922 | EPI3262923 |
| B/Victoria | B/Myanmar/16M525/2016 isolated | 2016/8/29 | EPI_ISL_19107993 | EPI3263035 | EPI3263036 | EPI3263037 | EPI3263038 | EPI3263039 | EPI3263040 | EPI3263041 | EPI3263042 |
| B/Victoria | B/Myanmar/16M564/2016 isolated | 2016/9/15 | EPI_ISL_19107994 | EPI3263043 | EPI3263044 | EPI3263045 | EPI3263046 | EPI3263047 | EPI3263048 | EPI3263049 | EPI3263050 |
| B/Victoria | B/Myanmar/19M056/2019 isolated | 2019/7/8 | EPI_ISL_19107995 | EPI3263051 | EPI3263052 | EPI3263053 | EPI3263054 | EPI3263055 | EPI3263056 | EPI3263057 | EPI3263058 |
| B/Victoria | B/Myanmar/19M076/2019 isolated | 2019/7/15 | EPI_ISL_19107996 | EPI3263059 | EPI3263060 | EPI3263061 | EPI3263062 | EPI3263063 | EPI3263064 | EPI3263065 | EPI3263066 |
| B/Victoria | B/Myanmar/19M105/2019 isolated | 2019/7/26 | EPI_ISL_19110272 | EPI3268170 | EPI3268171 | EPI3268172 | EPI3268173 | EPI3268174 | EPI3268175 | EPI3268176 | EPI3268177 |
| B/Victoria | B/Myanmar/19M112/2019 isolated | 2019/8/2 | EPI_ISL_19108180 | EPI3263115 | EPI3263116 | EPI3263117 | EPI3263118 | EPI3263119 | EPI3263120 | EPI3263121 | EPI3263122 |
| B/Victoria | B/Myanmar/19M116/2019 isolated | 2019/8/2 | EPI_ISL_19110273 | EPI3268178 | EPI3268179 | EPI3268180 | EPI3268181 | EPI3268182 | EPI3268183 | EPI3268184 | EPI3268185 |
| B/Victoria | B/Myanmar/19M123/2019 isolated | 2019/8/5 | EPI_ISL_19110274 | EPI3268186 | EPI3268187 | EPI3268188 | EPI3268189 | EPI3268183 | EPI3268191 | EPI3268192 | EPI3268193 |
| B/Victoria | B/Myanmar/19M155/2019 isolated | 2019/8/16 | EPI_ISL_19110275 | EPI3268194 | EPI3268195 | EPI3268196 | EPI3268197 | EPI3268198 | EPI3268201 | EPI3268199 | EPI3268200 |
| B/Victoria | B/Myanmar/19M156/2019 isolated | 2019/8/16 | EPI_ISL_19108181 | EPI3263123 | EPI3263124 | EPI3263125 | EPI3263126 | EPI3263127 | EPI3263128 | EPI3263129 | EPI3263130 |
| B/Victoria | B/Myanmar/19M164/2019 isolated | 2019/8/23 | EPI_ISL_19110344 | EPI3268202 | EPI3268203 | EPI3268204 | EPI3268205 | EPI3268206 | EPI3268207 | EPI3268208 | EPI3268209 |
| B/Victoria | B/Myanmar/19M167/2019 isolated | 2019/8/30 | EPI_ISL_19110345 | EPI3268210 | EPI3268211 | EPI3268212 | EPI3268213 | EPI3268214 | EPI3268215 | EPI3268216 | EPI3268217 |
| B/Victoria | B/Myanmar/19M310/2019 isolated | 2019/7/17 | EPI_ISL_19108153 | EPI3263067 | EPI3263068 | EPI3263069 | EPI3263070 | EPI3263071 | EPI3263072 | EPI3263073 | EPI3263074 |
| B/Victoria | B/Myanmar/19M313/2019 isolated | 2019/7/31 | EPI_ISL_19108172 | EPI3263074 | EPI3263075 | EPI3263076 | EPI3263077 | EPI3263078 | EPI3263079 | EPI3263080 | EPI3263081 |

Abbreviations: PB2, polymerase basic 2; PB1, polymerase basic 1; PA, polymerase acid; HA, hemagglutinin; NP, nucleocapsid protein; NA, neuraminidase; MP, matrix protein; NS, nonstructural protein.

**Supplementary Table 4. List of strain names and GISAID accession numbers for all eight segments of influenza B viruses sequenced in this study.**

| Lineage | Strain name | Collection Date  (Year/Month/Date) | ID | PB2 | PB1 | PA | HA | NP | NA | MP | NS |
| --- | --- | --- | --- | --- | --- | --- | --- | --- | --- | --- | --- |
| B/Victoria | B/Myanmar/19M314/2019 isolated | 2019/7/31 | EPI_ISL_19108176 | EPI3263099 | EPI3263100 | EPI3263101 | EPI3263102 | EPI3263103 | EPI3263104 | EPI3263105 | EPI3263106 |
| B/Victoria | B/Myanmar/19M335/2019 isolated | 2019/9/11 | EPI_ISL_19108179 | EPI3263107 | EPI3263108 | EPI3263109 | EPI3263110 | EPI3263111 | EPI3263112 | EPI3263113 | EPI3263114 |
| B/Yamagata | B/Myanmar/17M246/2017 isolated | 2017/9/4 | EPI_ISL_19110346 | EPI3268218 | EPI3268219 | EPI3268220 | EPI3268221 | EPI3268222 | EPI3268223 | EPI3268224 | EPI3268225 |
| B/Yamagata | B/Myanmar/17M249/2017 isolated | 2017/9/9 | EPI_ISL_19110347 | EPI3268226 | EPI3268227 | EPI3268228 | EPI3268229 | EPI3268230 | EPI3268231 | EPI3268232 | EPI3268233 |
| B/Yamagata | B/Myanmar/17M250/2017 isolated | 2017/9/9 | EPI_ISL_19110348 | EPI3268234 | EPI3268235 | EPI3268236 | EPI3268237 | EPI3268238 | EPI3268239 | EPI3268240 | EPI3268241 |
| B/Yamagata | B/Myanmar/17M275/2017 isolated | 2017/9/30 | EPI_ISL_19110352 | EPI3268242 | EPI3268243 | EPI3268244 | EPI3268245 | EPI3268246 | EPI3268247 | EPI3268248 | EPI3268249 |
| B/Yamagata | B/Myanmar/17M279/2017 isolated | 2017/10/2 | EPI_ISL_19110606 | EPI3268250 | EPI3268251 | EPI3268252 | EPI3268253 | EPI3268254 | EPI3268255 | EPI3268256 | EPI3268257 |
| B/Yamagata | B/Myanmar/17M289/2017 isolated | 2017/10/7 | EPI_ISL_19110607 | EPI3268258 | EPI3268259 | EPI3268260 | EPI3268261 | EPI3268262 | EPI3268263 | EPI3268264 | EPI3268265 |
| B/Yamagata | B/Myanmar/17M290/2017 isolated | 2017/10/7 | EPI_ISL_19110608 | EPI3268266 | EPI3268267 | EPI3268268 | EPI3268269 | EPI3268270 | EPI3268271 | EPI3268272 | EPI3268273 |
| B/Yamagata | B/Myanmar/18M006/2018 isolated | 2018/7/20 | EPI_ISL_19122588 | EPI3271292 | EPI3271293 | EPI3271294 | EPI3271295 | EPI3271296 | EPI3271297 | EPI3271298 | EPI3271299 |
| B/Yamagata | B/Myanmar/18M011/2018 isolated | 2018/7/23 | EPI_ISL_19122589 | EPI3271300 | EPI3271301 | EPI3271302 | EPI3271303 | EPI3271304 | EPI3271305 | EPI3271306 | EPI3271307 |
| B/Yamagata | B/Myanmar/18M015/2018 isolated | 2018/7/28 | EPI_ISL_19122590 | EPI3271308 | EPI3271309 | EPI3271310 | EPI3271311 | EPI3271312 | EPI3271313 | EPI3271314 | EPI3271315 |
| B/Yamagata | B/Myanmar/18M045/2018 isolated | 2018/8/6 | EPI_ISL_19122591 | EPI3271316 | EPI3271317 | EPI3271318 | EPI3271319 | EPI3271320 | EPI3271321 | EPI3271322 | EPI3271323 |
| B/Yamagata | B/Myanmar/18M083/2018 isolated | 2018/8/13 | EPI_ISL_19122592 | EPI3271324 | EPI3271325 | EPI3271326 | EPI3271327 | EPI3271328 | EPI3271329 | EPI3271330 | EPI3271331 |
| B/Yamagata | B/Myanmar/18M101/2018 isolated | 2018/8/17 | EPI_ISL_19122593 | EPI3271332 | EPI3271333 | EPI3271334 | EPI3271335 | EPI3271336 | EPI3271337 | EPI3271338 | EPI3271339 |
| B/Yamagata | B/Myanmar/18M116/2018 isolated | 2018/8/20 | EPI_ISL_19122594 | EPI3271340 | EPI3271341 | EPI3271342 | EPI3271343 | EPI3271344 | EPI3271345 | EPI3271346 | EPI3271347 |
| B/Yamagata | B/Myanmar/18M139/2018 isolated | 2018/8/24 | EPI_ISL_19122595 | EPI3271348 | EPI3271349 | EPI3271350 | EPI3271351 | EPI3271352 | EPI3271353 | EPI3271354 | EPI3271355 |
| B/Yamagata | B/Myanmar/18M148/2018 isolated | 2018/8/27 | EPI_ISL_19122596 | EPI3271356 | EPI3271357 | EPI3271358 | EPI3271359 | EPI3271360 | EPI3271361 | EPI3271362 | EPI3271363 |
| B/Yamagata | B/Myanmar/18M156/2018 isolated | 2018/8/31 | EPI_ISL_19122597 | EPI3271364 | EPI3271365 | EPI3271366 | EPI3271367 | EPI3271368 | EPI3271369 | EPI3271370 | EPI3271371 |
| B/Yamagata | B/Myanmar/18M169/2018 isolated | 2018/9/3 | EPI_ISL_19122598 | EPI3271372 | EPI3271373 | EPI3271374 | EPI3271375 | EPI3271376 | EPI3271377 | EPI3271378 | EPI3271379 |
| B/Yamagata | B/Myanmar/18M190/2018 isolated | 2018/9/7 | EPI_ISL_19122599 | EPI3271380 | EPI3271381 | EPI3271382 | EPI3271383 | EPI3271384 | EPI3271385 | EPI3271386 | EPI3271387 |
| B/Yamagata | B/Myanmar/18M227/2018 isolated | 2018/9/14 | EPI_ISL_19122600 | EPI3271388 | EPI3271389 | EPI3271390 | EPI3271391 | EPI3271392 | EPI3271393 | EPI3271394 | EPI3271395 |
| B/Yamagata | B/Myanmar/18M278/2018 isolated | 2018/9/30 | EPI_ISL_19122601 | EPI3271396 | EPI3271397 | EPI3271398 | EPI3271399 | EPI3271400 | EPI3271401 | EPI3271402 | EPI3271403 |
| B/Yamagata | B/Myanmar/18M305/2018 isolated | 2018/10/12 | EPI_ISL_19122602 | EPI3271404 | EPI3271405 | EPI3271406 | EPI3271407 | EPI3271408 | EPI3271409 | EPI3271410 | EPI3271411 |

Abbreviations: PB2, polymerase basic 2; PB1, polymerase basic 1; PA, polymerase acid; HA, hemagglutinin; NP, nucleocapsid protein; NA, neuraminidase; MP, matrix protein; NS, nonstructural protein.
